# Identification and expression profile analysis of the sucrose phosphate synthase gene family in *Litchi chinensis* Sonn.

**DOI:** 10.7717/peerj.4379

**Published:** 2018-02-15

**Authors:** Dan Wang, Jietang Zhao, Bing Hu, Jiaqi Li, Yaqi Qin, Linhuan Chen, Yonghua Qin, Guibing Hu

**Affiliations:** State Key Laboratory for Conservation and Utilization of Subtropical Agro-bioresources/Key Laboratory of Biology and Genetic Improvement of Horticultural Crops-South China, Ministry of Agriculture, College of Horticulture, South China Agricultural University, Guangzhou, China; Guangdong Litchi Engineering Research Center, College of Horticulture, South China Agricultural University, Guangzhou, China

**Keywords:** Sucrose phosphate synthase, *Litchi chinensis* Sonn., Gene expression, Aril, Sugar accumulation

## Abstract

Sucrose phosphate synthase (SPS, EC 2.4.1.14) is a key enzyme that regulates sucrose biosynthesis in plants. SPS is encoded by different gene families which display differential expression patterns and functional divergence. Genome-wide identification and expression analyses of *SPS* gene families have been performed in Arabidopsis, rice, and sugarcane, but a comprehensive analysis of the *SPS* gene family in *Litchi chinensis* Sonn. has not yet been reported. In the current study, four *SPS* gene (*LcSPS1*,* LcSPS2*, *LcSPS3*, and *LcSPS4*) were isolated from litchi. The genomic organization analysis indicated the four litchi *SPS* genes have very similar exon-intron structures. Phylogenetic tree showed *LcSPS1*-*4* were grouped into different SPS families (*LcSPS1* and *LcSPS2* in A family, *LcSPS3* in B family, and *LcSPS4* in C family). *LcSPS1* and *LcSPS4* were strongly expressed in the flowers, while *LcSPS3* most expressed in mature leaves. RT-qPCR results showed that *LcSPS* genes expressed differentially during aril development between cultivars with different hexose/sucrose ratios. A higher level of expression of *LcSPS* genes was detected in Wuheli, which accumulates higher sucrose in the aril at mature. The tissue- and developmental stage-specific expression of *LcSPS1*-*4* genes uncovered in this study increase our understanding of the important roles played by these genes in litchi fruits.

## Introduction

Litchi (*Litchi chinensis* Sonn.) belongs to the Sapindaceae family and is an important evergreen fruit crop grown in the tropical and subtropical regions of the world. The edible portion of litchi fruit is semi-translucent to white aril (flesh), which accumulates sugars that account for 15–20% of the fresh mass. Sugar content and other compounds in the aril of litchi determine the fruit quality and flavor. In litchi, sucrose, fructose, and glucose are the major sugar in the aril, and the sugar content varies considerably among cultivars (Paull et al., 1984; Wang et al., 2006; Yang et al., 2013; Wu et al., 2016). The aril initiates from the funicle, where sucrose phloem unloading through symplastic pathway. Recent study reveals evidence for an apoplasmic post-phloem sucrose transport from the funicle to the aril (Wang et al., 2015). Sucrose enters the sink cells as sucrose or as hexoses after hydrolysis by cell wall invertase (Vimolmangkang et al., 2016). The transported sugars in sink cells are either stored or metabolized. However, our knowledge of the mechanisms underlying sugar accumulation in litchi has not been reported so far. Identifying key genes involved in sugar metabolism in litchi could be beneficial for elucidating the molecular mechanism of sucrose accumulation.

Sucrose phosphate synthase (SPS; EC 2.4.1.14), a key enzyme of sucrose synthesis, catalyzes the conversion of Fructose-6-Phosphate and UDP-glucose into Sucrose-6-Phosphate, which is then hydrolysed by sucrose-phosphatase (SPP) to sucrose (Lunn & MacRae, 2003). SPS activity has been shown to be linked with plant growth and yield (Causse et al., 1995; Prioul et al., 1999). In rice, the quantitative trait locus for plant height appeared to coincide with the *OsSPS1* locus, and transgenic rice had higher SPS activity and grew taller (Ishimaru, Ono & Kashiwagi, 2004). SPS has also been proposed to be a controlling factor in regulation of sucrose synthesis or accumulation in source leaves and sucrose-storing sink tissues (Kerr & Huber, 1987; Miron & Schaffer, 1991; Komatsu et al., 1996). Zhu, Komor & Moore (1997) have shown that sucrose accumulation in the sugarcane stem is dependent on the activity of SPS. SPS might be used as a biochemical marker of high sucrose accumulation in sugarcane (Grof et al., 2007). Similar results have also been observed in tomato and muskmelon fruit (Lingle & Dunlap, 1987; Miron & Schaffer, 1991). In litchi, the relationship between sugar accumulation and SPS activity or gene expression is not clear (Yang et al., 2013).

Recent studies have shown that SPS is encoded by a multi-gene family in both dicotyledonous and monocotyledonous plants (Lunn & MacRae, 2003). There are 4 and 5 members of SPS genes in the Arabidopsis and rice genomes, respectively (Langenkämper et al., 2002; Okamura et al., 2011). Langenkämper et al. (2002) carried out phylogenetic analysis of known SPS genes in dicotyledonous plants and divided SPS genes into three families designated A, B, and C. But Castleden et al. (2004) found a novel and distinctive D family in wheat and other monocotyledonous plants in addition to the previously described A, B, and C gene families. The D family could be further divided into two subfamilies (Lutfiyya et al., 2007). Expression and functions of different *SPS* genes vary among different families in different plants and very little data is available to understand the functional features of *SPS* genes in the same plant (Wang et al., 2013).

In our previous study, a *LcSPS* (JQ773416) gene was isolated and expression analysis was carried out in the arils of different litchi cultivars (Yang et al., 2013). In this paper, we report on the characterization of the four SPS genes in litchi and analyze their expression levels during the aril development.

## Materials and Methods

### Plant materials

*Litchi chinensis* cvs. Feizixiao (FZX) and Wuheli (WHL) trees were grown in the orchard of South China Agricultural University, Guangzhou, China. The selected trees were under the same integrated orchard management practices. Male flowers, female flowers, young leaves, mature leaves, young stems, young roots were collected from FZX. For tissue-specific gene expression, the pericarps, seeds and arils of mature fruits of FZX were used. For gene expression in fruit development, fruits were sampled between May 1 and June 30, 2016, at intervals of five days after anthesis (DAA) until maturity. The samples were taken to the laboratory immediately and arils were separated and frozen immediately in liquid nitrogen and stored at −80 °C until used.

### Extraction and determination of sugars

Soluble sugars were extracted and determined according to the method as described by Yang et al. (2013).

### RNA extraction and cDNA synthesis

Total RNA was extracted using the RNAprep Pure Plant Kit (TIANGEN, Beijing, China) according to the manufacturer’s instructions. RNase-free DNase I (TIANGEN, Beijing, China) was used in the extraction process to remove DNA contamination. Total RNA was spectrophotometrically quantified and then electrophoretically checked on 1.0% agarose gels to verify integrity. Thereafter, approximately 1 µg of total RNA per sample was reverse-transcribed using RevertAid First Strand cDNA Synthesis Kit (Thermo Fisher, Waltham, MA, USA) following the manufacturer’s instructions. All cDNA samples were stored at −20 °C before used as template in gene cloning and reverse transcription quantitative real-time PCR (RT-qPCR) analysis.

### *LcSPSs* cDNA cloning and genomic sequence assembly

Based on the litchi reference genome sequences and gene functional annotations (G Hu, 2016, unpublished data). Four *LcSPS* genes were obtained and the gene-specific primers (Table S1) were designed using the Primer 5.0 program. The cDNA of FZX aril was used as templates in each PCR reaction with the KOD-PLUS-NEO Taq polymerase (TOYOBO, Japan) following the manufacturer’s instructions. Amplification products were cloned into the pMD19-T cloning vector (TaKaRa, Dalian, China) and then transformed into *Escherichia coli* competent cells (DH5a) for sequencing.

Genomic sequences, genomic DNA size and genome locations of *LcSPS* genes were obtained from the Litchi Genome database (G Hu, 2016, unpublished data). Exon/intron structures were analyzed by comparing the cDNA sequences and their genomic DNA sequences using the Splign online tool (https://www.ncbi.nlm.nih.gov/sutils/splign/splign.cgi). The schematic of genetic structure was drawn by DNAMAN 6.0 software and modified by Adobe Illustrator CS5 software.

### Sequence analysis

The amino acid sequence was predicted by the translate tool of ExPASy (https://web.expasy.org/translate/). The basic physical and chemical characteristics including the number of amino acids, molecular weight, and predicted theoretical isoelectric point were calculated by using the online ProtParam tool (http://www.expasy.org/tools/protparam.html).

Multiple sequences alignment was performed by ClustalX software. The phylogenetic tree was constructed by MEGA 7.0 using neighbor-joining (NJ) method and bootstrap methods with 1,000 replications (Kumar, Stecher & Tamura, 2016).

### RT-qPCR analysis

RT-qPCR was conducted to determine the expression profile for each member of the *LcSPS* genes using various tissues and developmental stages of arils. Amplification was carried out using AceQ™ qPCR SYBR Green Master Mix (Vazyme, China) in 20 mL volume. And the reactions were run in Applied Biosystems 7500 Real-Time PCR System (Life Technologies Corporation, Beverly, MA, USA). The primers were designed by Primer 3 (http://primer3.ut.ee/) and listed in Table S2. All qPCR reactions were normalized using the Ct value corresponding to the *LcACTIN* gene (HQ615689). Relative expression levels of candidate genes were calculated with the formula 2^−ΔΔCT^ (Livak & Schmittgen, 2001). All biological replicates were measured in triplicate.

### Statistical analysis

Data were expressed as mean ± standard error (SE) using the Excel 2003 or SigmaPlot software version 12.5 (Systat Software Inc., San Jose, CA, USA).

## Results

### Sugar contents and composition during aril development

**Figure 1:**
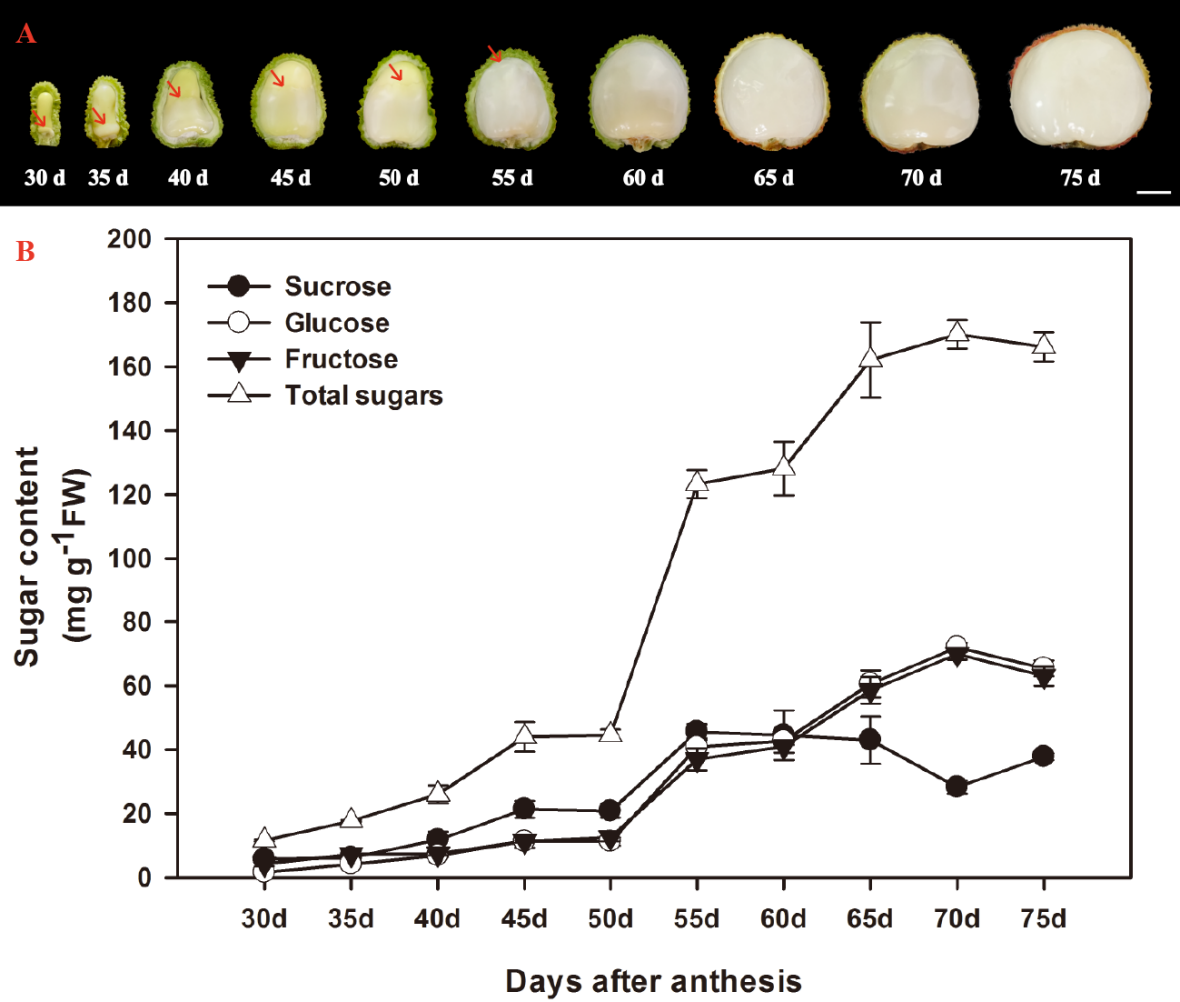
Changes in aril growth (A) and the contents of sucrose, glucose, fructose, and total sugars (B) of *Litchi chinensis* Sonn. cv. Feizixiao during fruit development. The vertical bars represent the standard error of three replicates. The arrowhead refers to the arils.

According to Huang & Xu (1983), the ontogeny of litchi fruit could be divided into two stages: the seed coat and pericarp (peel) grow first, then the cotyledon and aril grow. The aril in the litchi fruit initiates from the funicle about 7 DAA on the opposite site of micropyle and appears as late as about 30 DAA on the micropylar site (Huang & Xu, 1983). In the present study, the aril in the fruit of FZX were appeared around the funicle 30 DAA, and it grew upwards, gradually enclosed the seed (Fig. 1A). The aril is white to translucent and contains around 15–20% dry mass.

The developmental changes in sucrose, glucose, fructose, and total sugars were analyzed in arils of FZX (Fig. 1B). Sucrose, glucose and fructose are the major sugar in aril of litchi. The sugar content in the arils increased with the aril growth. The increase of sugar in the arils began to accelerate after 50 DAA and total sugar attained to 170.148 mg g^−1^ FW in 70 DAA. In FZX, an increase in sucrose was observed before 55 DAA but this trend declined as fruit matured; the content of glucose and fructose increased steadily at the same time, resulting in a distinct increase in hexose/sucrose ratios during the late aril growth stage.

### Litchi *SPS* genes

We identified four *SPS* genes in litchi genome, which were nominated as *LcSPS1*-*4*, and full-length cDNA of each gene was cloned by PCR using gene-specific primers. *LcSPS1* had been isolated in our previously study (Yang et al., 2013). The cDNA and deduced amino acid sequences of *LcSPS2*-*4* were deposited in GenBank (*LcSPS2*, MG832657; *LcSPS3*, MG832658; *LcSPS4*, MG832659) and also listed in the Supplemental Dataset File. Bioinformatics analysis by using the online ProtParam tool was presented in Table 1. The deduced proteins encoded by these *LcSPS* genes contain 1,023–1,069 amino acids (predicted 115.3 to 120.1 kDa in molecular weight) with their isoelectric points calculated ranging from 6.10 to 6.74 (Table 1).

**Table 1:** The information and characteristics of *LcSPS* genes.

Name	Gene ID	Gene location	Genomic DNA size (bp)	ORF (bp)	No. of amino acids	Predicted Mw (kDa)	Theoretical pI
*LcSPS1*	Litchi_GLEAN_10026856	scaffold681:232024-238696(+)	6,673	3,174	1,057	118.5	6.11
*LcSPS2*	Litchi_GLEAN_10011902	scaffold1604:157081-161233(+)	5,416	3,156	1,051	117.7	6.39
*LcSPS3*	Litchi_GLEAN_10015418	scaffold2228:68770-76017(+)	7,248	3,210	1,069	120.1	6.1
*LcSPS4*	Litchi_GLEAN_10017506	scaffold1659:94376-102551(−)	8,176	3,072	1,023	115.3	6.74

**Figure 2:**
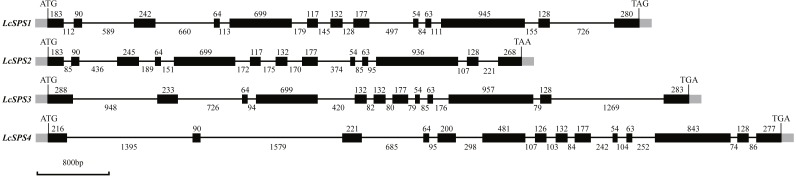
Schematic representation of the four *LcSPS* gene structures. Black and gray boxes represent exons within coding regions and untranslated regions, respectively. The lines connecting them represent introns. Numbers above boxes or under lines denote the sizes (bp) of corresponding exons or introns, respectively.

The four *SPS* genes in litchi were located on four scaffolds, and each scaffold contained only one gene (Table 1). Sequence comparison revealed that the *LcSPS* genes share a high sequence homology at the amino acid level (53.67% to 77.59% identity) (Table 2). *LcSPS1* shared much higher levels of identity of the amino acid with *LcSPS2* compared with the other paralogs. Moreover, the genomic organization of *LcSPS* genes was determined by comparing the cDNA sequences with genomic DNA. As shown in Fig. 2, the four *LcSPS* genes had 12–14 exons and 11–13 introns. The four litchi *SPS* genes have very similar exon-intron structures, with the *LcSPS1* and *LcSPS2* genes containing 12 introns almost at the same positions in the coding regions (Fig. 2). The *LcSPS3* and *LcSPS4* genes differ with respect to intron loss or gain events. *LcSPS3* lacks the equivalent of first intron, resulting in formation of a larger exon 1. In addition, the equivalent of exon 5 of *LcSPS4* gene is inserted by a 298 bp intron. As a consequence, the *LcSPS4* gene has an additional exon that is not observed in *LcSPS1*-*3*.

**Table 2:** Identity matrix of the four *LcSPS* nucleotide/amino acid sequences.

Score of identity for nucleotide sequences (%)	Score of identity for amino acid sequences (%)			
	*LcSPS1*	*LcSPS2*	*LcSPS3*	*LcSPS4*
*LcSPS1*	–	77.59^a^	57.79^a^	53.67^a^
*LcSPS2*	77.2^b^	–	58.16^a^	54.15^a^
*LcSPS3*	62.52^b^	64.85^b^	–	58.22^a^
*LcSPS4*	63.07^b^	62.38^b^	65.00^b^	–

**Notes.**

a For identity of amino acid sequences.

b For identity of nucleotide sequences.

### Sequence alignments and phylogenetic analysis

**Figure 3:**
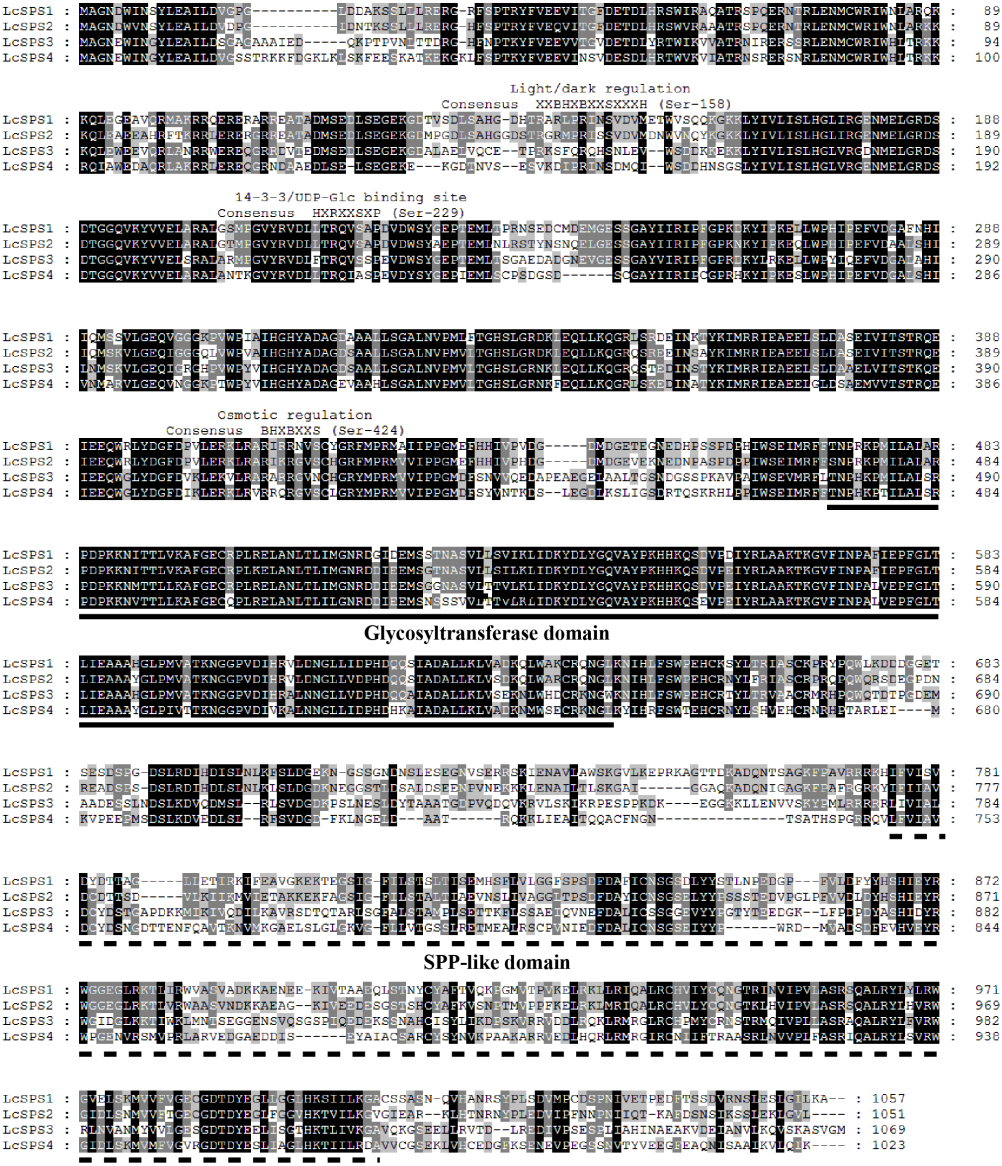
Alignment analysis of deduced amino acid sequences of four *LcSPS* proteins. Sequence alignment analysis was performed using the multiple alignment program of the ClustalX software. Identical amino acids are shaded, and gaps are indicated by dots. The conserved characteristic glycosyltransferase domain (black solid line) and SPP-like domain (black dotted line) domain were marked out. The positions of the phosphorylation sites involved in light-dark regulation (Ser-158), 14-3-3 protein binding (Ser-229), and osmotic stress activation (Ser-424) of the plant SPS are marked out by the words.

Multiple sequence alignment of the four *LcSPS* gene was performed with ClustalX software (Fig. 3). The four members share the two characteristic functional domains of *SPS* genes, a glucosyltransferase domain (N-domain) and a SPP-like domain (C-domain). Apart from two domains on SPS, three regulatory phosphorylation sites involved in light/dark regulation, 14-3-3 protein binding, and osmotic stress activation were also conserved in in *LcSPS1* and *LcSPS2*. However, there is no 14-3-3 protein binding site in *LcSPS4*, and osmotic stress activation site in *LcSPS3*.

**Figure 4:**
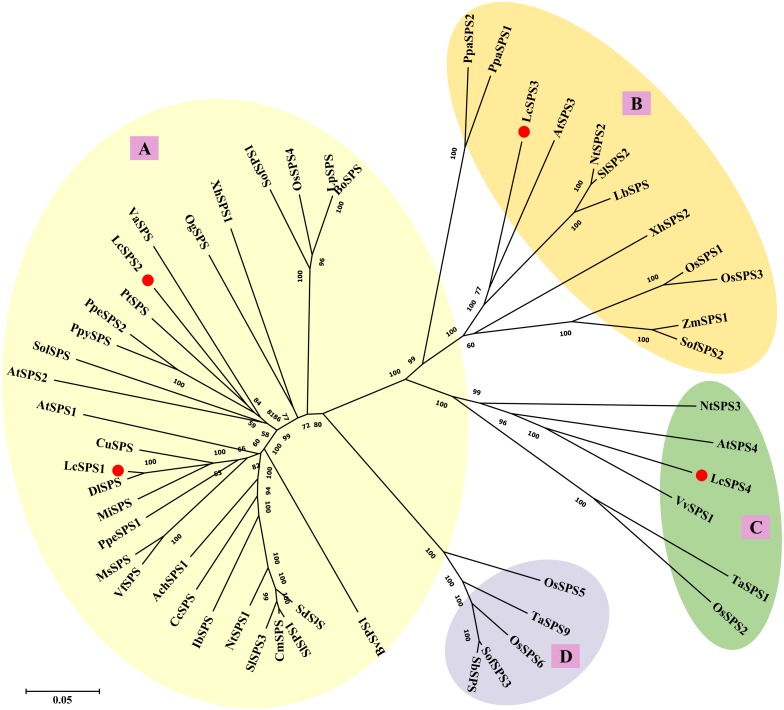
Phylogenetic analysis of the SPS proteins from litchi and other plants. At, *Arabidopsis thaliana*; Ach, *Actinidia Chinensis*; Bo, *Bambusa oldhamii*; Bv, *Beta vulgaris*; Cc, *Coffea canephora*; *Cm*, *Cucumis melo*; Cu, *Citrus unshiu*;* Dl*, *Dimocarpus longan*; Ib, *Ipomoea batatas*; Lc, *Litchi chinensis*; Lb, *Lycium barbarum*; Lp, *Lolium perenne*; Ms, *Medicago sativa*; Mi, *Mangifera indica*; Nt, *Nicotiana tabacum*; Os, *Oryza sativa*; Og, *Oncidium Goldiana*; Pt, *Populus trichocarpa*; Ppa, *Physcomitrella patens*; Ppe, *Prunus persica*; Ppy, *Pyrus pyrifolia*; Sof, *Saccharum officinarum*; St, *Solanum tuberosum*; Sb, *Sorghum bicolor*; Sl, *Solanum lycopersicum*; Sol, *Spinacia oleracea*; Ta, *Triticum aestivum*; Va, *Viscum album subsp. album*; Vf, *Vicia faba var. minor*; Vv, *Vitis vinifera*; Xh, *Xerophyta humilis*; Zm, *Zea mays*. The accession numbers are listed in Table S3.

To better understand the evolutionary relationships among the SPS genes of litchi and other plant species, 53 amino acid sequences from 32 species were used to construct the phylogenetic tree. As shown in Fig. 4, plant *SPS* genes could be divided into four distinct clusters: A, B, C and D family as previously studies (Castleden et al., 2004). *LcSPS1* and *LcSPS2* were classified into A family, while *LcSPS3* and *LcSPS4* were classified into B and C families, respectively. *LcSPS3* had a closer relationship to Arabidopsis *AtSPS3* within the B family; however, *LcSPS4* was more closely related to grapevine *VvSPS1* in the C family.

### Tissue-specific expression of *LcSPS* genes

**Figure 5:**
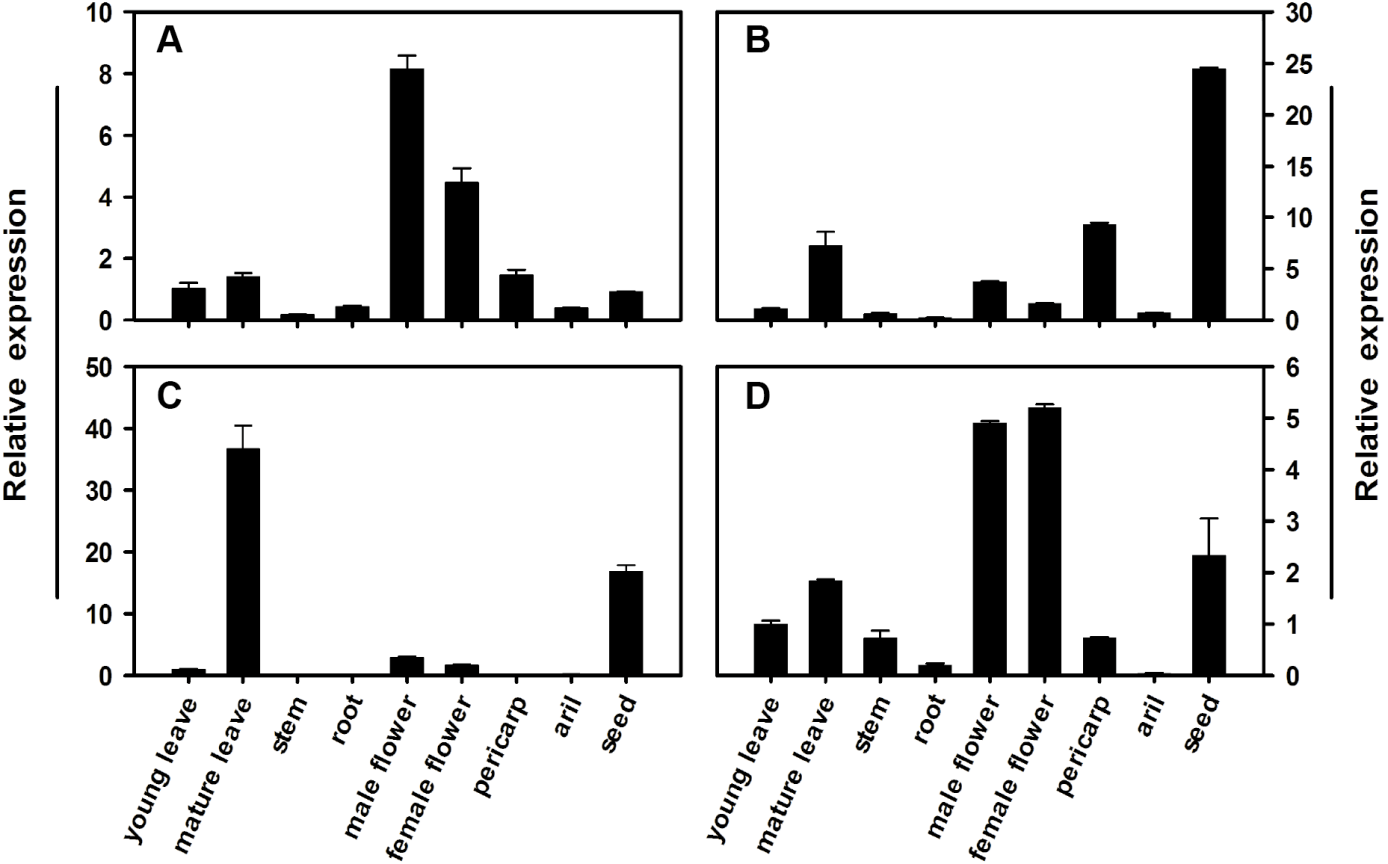
Changes in the expression of *LcSPS1* (A), *LcSPS2* (B), *LcSPS3* (C), and *LcSPS4* (D) as determined by RT-qPCR in FZX different tissues. *LcActin* gene was used to normalize gene expression under identical conditions. The vertical bars represent the standard error of three replicates.

To determine the expression profile for each member of the litchi *SPS* gene family, qRT-PCR analysis was conducted using different tissues and pericarp, aril, and seed from mature fruit in FZX. Figure 5 illustrates the relative mRNA abundance of each *LcSPS* gene. There is considerable variation in the expression patterns of *LcSPS* genes in different tissues. *LcSPS1* and *LcSPS4* were most strongly expressed in the flowers (Figs. 5A and 5D). High level expression of *LcSPS2* was detected in seeds (Fig. 5B). In contrast, *LcSPS3* was most strongly expressed in mature leaves, and with some expression also found in seeds (Fig. 5C).

### Expression level of *LcSPS* genes in developmental aril

**Figure 6:**
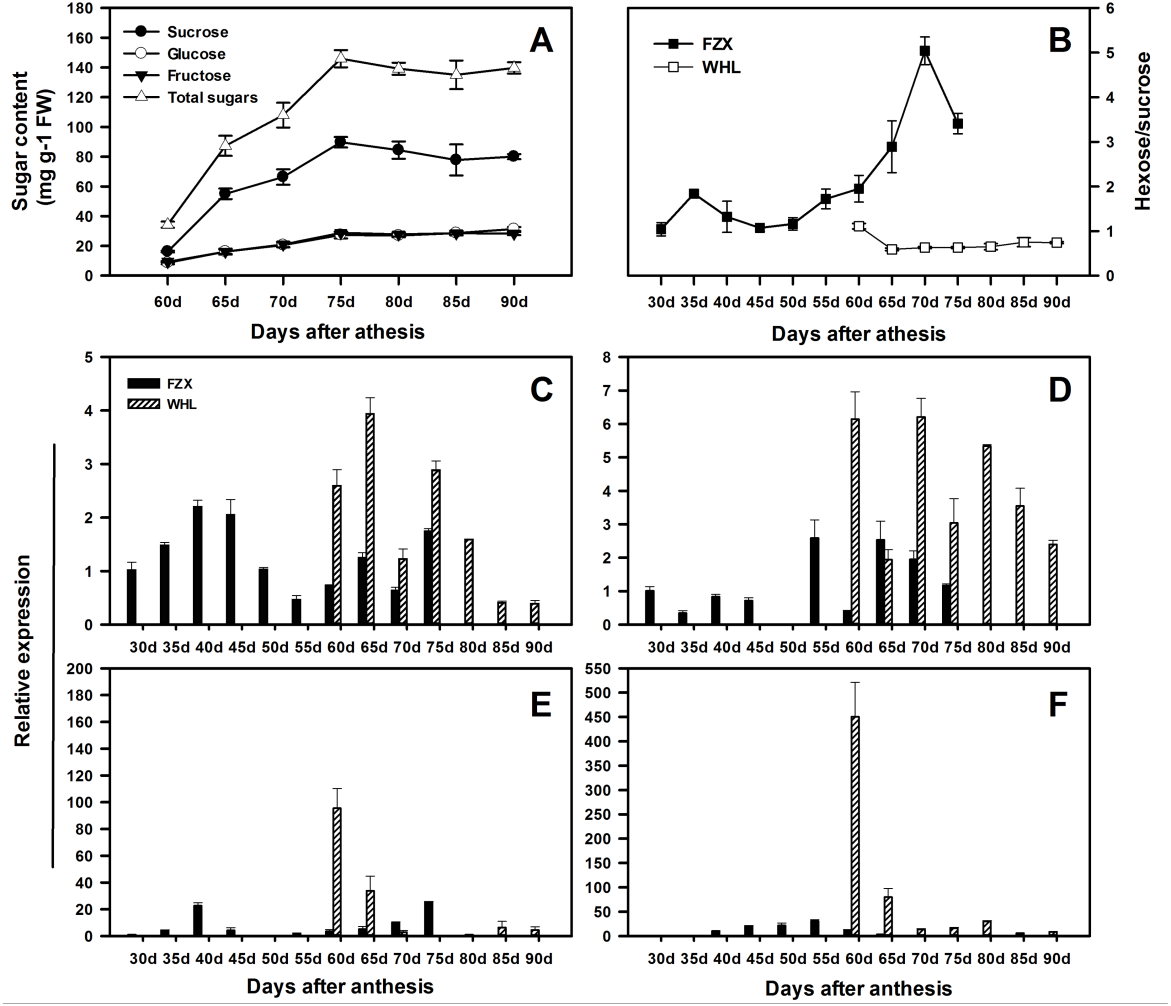
Changes in sugar contents and the expression of *LcSPS1*, *LcSPS2*, *LcSPS3* and *LcSPS4* at different developmental stage of litchi aril. (A) The developmental changes in sucrose, glucose, fructose, and total sugars were analyzed in arils of WHL. (B) Comparision of the hexose/sucrose ratios changes between FZX and WHL during aril development. The expression of *LcSPS1* (C), *LcSPS2* (D), *LcSPS3* (E), and *LcSPS4* (F) were determined by RT-qPCR in FZX and WHL aril during fruit development. *LcActin* gene was used to normalize gene expression under identical conditions. The vertical bars represent the standard error of three replicates.

In order to speculate the functions of *LcSPS* genes in the regulation of sucrose accumulation in litchi aril, the temporal expression features in varieties with different sucrose contents were analyzed using RT-qPCR. According to Yang et al. (2013), there is significant difference in hexose/sucrose ratio in the arils of litchi cultivars at maturity. In WHL, sucrose and hexose increased steadily during the whole aril growth stage (Fig. 6A), resulting in a distinct decrease in hexose/sucrose ratios when compared with FZX (Fig. 6B).

The RT-qPCR results showed that there was considerable variation in the expression patterns of *LcSPS* genes during litchi aril development and between cultivars (Figs. 6C–6F). In FZX, the expression of *LcSPS1*-*3* was low throughout aril growth, except an increase of *LcSPS3* on 40 DAA; however, a significant increase was observed on the expression of *LcSPS4* during aril growth, which was correlation with the sucrose accumulation in the aril (Fig. 1B). Compared with FZX, all the *LcSPS* genes in WHL exhibited higher expression during the late stage of aril development, especially the *LcSPS4* gene (Fig. 6F). Since WHL accumulates higher sucrose in the aril at mature, the higher level of expression of *LcSPS* genes at the late stage of aril development probably help ensure stable sucrose synthesis in aril.

## Discussion

*SPS* gene was first cloned from maize (Worrell et al., 1991). Subsequently, *SPS* genes have been cloned from over 20 plant species (Lunn & MacRae, 2003; Castleden et al., 2004). There is evidence that more than one *SPS* gene exists in higher plants (Komatsu et al., 1996; Langenkämper et al., 2002). With the large-scale genomic sequencing, more *SPS* gene family members on a genome-wide level were analyzed (Jiang et al., 2015). In the present study, four *LcSPS* genes were isolated and characterized from litchi, which is consistent with the findings that most of plants encode 3–5 *SPS* genes. However, there is up to 7 *SPS* genes identified in apple, maize, and soybean (Castleden et al., 2004; Jiang et al., 2015). The different expansion of *SPS* gene family might explain that plant species encode different numbers of *SPS* genes (Jiang et al., 2015).

Castleden et al. (2004) carried out phylogenetic analysis of all the known *SPS* genes and found that they could cluster into four distinct families: A, B, C, and D. Family D is only found in the Poaceae (Castleden et al., 2004; Lutfiyya et al., 2007; Huang et al., 2017). Our results support plant *SPS* genes could be divided into four groups (Fig. 4) and no member belonging to family D was identified in litchi. Three families (A, B, and C) of the *LcSPS* genes exist in litchi. *LcSPS1* and *LcSPS2* belong to A family, which is the largest one consisting of most of the available sequences from both dicotyledonous and monocotyledonous plants. *LcSPS3* and *LcSPS4* were clustered into B and C families, respectively, indicating there is at least one *SPS* gene from each family. In the previous studies, it was shown that the expression patterns and functional features of *SPS* genes vary among different families from different plants (Reimholz et al., 1997; Huang et al., 2017). *LcSPS1*-*4* gene belonged to different family, suggesting functional divergence.

The spatial and temporal expression patterns of *SPS* genes vary among different families in different plants. However, only few studies focused on the comprehensive expression analysis of all the *SPS* gene families in a single plant species (Reimholz et al., 1997; Fung et al., 2003; Castleden et al., 2004; Okamura et al., 2011). In litchi, the expression profiles of the *SPS* gene family, except for *LcSPS1*, have not been investigated previously. In the present study, the other litchi *SPS* genes, *LcSPS2*, *LcSPS3* and *LcSPS4* were analyzed for the first time. In most of cases, the *LcSPS* genes were expressed in multiple tissues and expression levels varied among different tissues (Fig. 5). *LcSPS3* (family B) was strongly expressed in mature leaves, suggesting that *LcSPS3* is principally expressed in source tissues. *OsSPS1* in rice belonging to B family was also reported to be present in source tissues, particularly in leaf blades (Chávez-Bárcenas et al., 2000; Okamura et al., 2011). However, the expression level of *SofSPSB* (family B) in sugarcane is negligible in fully expanded leaves, indicating that *SofSPSB* is not involved in sucrose synthesis in source leaf (Huang et al., 2017). Further study is needed to investigate the expression patterns of B family *SPS* genes in more plant species to figure out whether it could be used as a source-specific gene. In addition, the mRNA level of *LcSPS4* (family C) was increasing during aril growth coinciding with sucrose accumulation (Fig. 6). In sugarcane, Huang et al. (2017) found the intensity of sucrose synthesis is controlled by regulating *SPSC* and *SPSA* genes expression. The role of SPS C in tobacco is postulated to participate in sucrose synthesis in the phase of starch mobilisation at night (Gibon et al., 2004). However, the exact roles of *LcSPS4* genes in sucrose synthesis during aril grow needs to be studied further.

The sugar composition in the aril of mature fruit varied widely among different litchi cultivars. FZX and WHL belong to hexose-prevalent type and sucrose-prevalent type, respectively, based on differences in hexose/sucrose ratios (Wang et al., 2006; Yang et al., 2013). In the previous study, Yang et al. (2013) reported that the sugar composition in litchi aril was dependent on the sucrose cleavage enzymes rather than the sucrose synthetic enzyme SPS. However, the expression levels of *LcSPS* genes in WHL were much higher than those in FZX. Indeed, sugar accumulation in fruit comprises a complex regulatory network (Ludewig & Flügge, 2013; Cirilli, Bassi & Ciacciulli, 2016). The transported sucrose in fruits is stored or broken down as hexoses. Sugar metabolism in higher plants comprises a complex regulatory network involving at least nine enzymes that contribute to the synthesis and degradation (Jiang et al., 2015). In fruits, sucrose re-synthesis is catalyzed mainly by SPS, which play important roles in sugar accumulation (Hubbard, Pharr & Huber, 1991). Higher activities and expression levels of SPS in WHL indicate the involvement of SPS in sucrose re-synthesis to help ensure stable sucrose accumulation in litchi aril with low hexose/sucrose ratios.

## Conclusions

In summary, four litchi *SPS* gene (*LcSPS1*, *LcSPS2*, *LcSPS3*, and *LcSPS4*) were isolated and characterized. Phylogenetic analysis showed *LcSPS1*-*4* were grouped into different SPS families and might play different roles in litchi. RT-qPCR results showed that *LcSPS1* and *LcSPS4* were strongly expressed in the flowers, while *LcSPS3* most expressed in mature leaves. *LcSPS* genes expressed differentially during aril development between cultivars with different hexose/sucrose ratios. Further functional studies should be undertaken to understand the roles of *LcSPS* genes during sugar accumulation in litchi.

##  Supplemental Information

10.7717/peerj.4379/supp-1 Table S1 Specific primers for the cloning of *LcSPS* genes in litchi

10.7717/peerj.4379/supp-2 Table S2 Gene-specific primers for RT-qPCR analysis

10.7717/peerj.4379/supp-3 Table S3 List of SPS proteins used for the construction of the phylogenetic tree

10.7717/peerj.4379/supp-4 Supplemental Information 1 The cDNA and deduced amino acid sequences of *LcSPS1*-*4*

10.7717/peerj.4379/supp-5 Data S1 Raw data of sugar content applied for data analyses and preparation for Figs. 1B, 6A and 6B

10.7717/peerj.4379/supp-6 Data S2 Raw data of gene expression applied for data analyses and preparation for Figs. 5, 6C–6F

## Abbreviations

Bp: base pair(s)
CDS: coding sequence
cDNA: DNA complementary to RNA
d: day(s)
DAA: days after anthesis
DNase: deoxyribonuclease
mg: milligrams
HPLC: high-performance liquid chromatography
kDa: kilodalton(s)
MW: molecular weight
ORF: open reading frame
PCR: polymerase chain reaction
pI: isoelectric point
RT-qPCR: reverse transcription quantitative real-time polymerase chain reaction
SPS: sucrose phosphate synthase
μg: microgram

## Additional Information and Declarations

### DNA Deposition

The following information was supplied regarding the deposition of DNA sequences: The sequences have been supplied as a Supplemental Dataset File.

### Data Availability

The following information was supplied regarding data availability: The raw data and the sequences have been supplied as Supplemental Dataset Files.
