# Supplementary material for: Identification and expression profile analysis of the sucrose phosphate synthase gene family in Litchi chinensis Sonn
Source: PeerJ. 2018 Feb 15;6:e4379. doi: 10.7717/peerj.4379 (PMC5816967; doi:10.7717/peerj.4379)
Supplement: Table S1 [file peerj-06-4379-s001.docx]

Table S1 Specific primers for the cloning of *LcSPS* genes in litchi

| **Primer Name** | **Sequence (5’-3’)** | **Length of**  **product (bp)** |
| --- | --- | --- |
| *LcSPS1*-F | ATGGCAGGAAACGACTGGATAAAC | 3174 |
| *LcSPS1*-R | GTAGGCCTTAAGAATTCCTAAACTCTC |  |
| *LcSPS2*-F | ATGGCGGGAAACGACTGGGTTA | 3156 |
| *LcSPS2*-R | TTAAAGAACCCCGAGTTTTTCCAATG |  |
| *LcSPS3*-F | ATGGCTGGCAACGAGTGG | 3210 |
| *LcSPS3*-R | TCACATTCCTACAGAAGCTTTGG |  |
| *LcSPS4*-F | ATGGCAGGAAATGAGTGGAT | 3072 |
| *LcSPS4*-R | TCACTTGATCTGAAGAACCTTTATAG |  |
