# Supplementary material for: Identification and expression profile analysis of the sucrose phosphate synthase gene family in Litchi chinensis Sonn
Source: PeerJ. 2018 Feb 15;6:e4379. doi: 10.7717/peerj.4379 (PMC5816967; doi:10.7717/peerj.4379)
Supplement: Table S2 [file peerj-06-4379-s002.docx]

Table S2 Gene-specific primers for RT-qPCR analysis

| **Primer Name** | **Sequence (5’-3’)** | **Length of**  **product (bp)** |
| --- | --- | --- |
| *LcACTIN*-F | ACCGTATGAGCAAGGAAATCACTG | 160 |
| *LcACTIN*-R | TCGTCGTACTCACCCTTTGAAATC |  |
| *QLcSPS1*-F | CTTTGATTCGTTGGGTAGC | 210 |
| *QLcSPS1*-R | TCCTGGTGCCATTTTGAC |  |
| *QLcSPS2*-F | CTCTGCTGCTCTTCTGTCTG | 163 |
| *QLcSPS2*-R | TAACTCCTCGGCTTCTATCC |  |
| *QLcSPS3*-F | GTGCTGCTCTTCTTTCTGGT | 157 |
| *QLcSPS3*-R | TCCTCTGCTTCTATCCTTCG |  |
| *QLcSPS4*-F | GTGGGCGTAAGAGGAGATAC | 149 |
| *QLcSPS4*-R | CTGCCTTCTGGGACTACATT |  |
