# Supplementary material for: Identification and expression profile analysis of the sucrose phosphate synthase gene family in Litchi chinensis Sonn
Source: PeerJ. 2018 Feb 15;6:e4379. doi: 10.7717/peerj.4379 (PMC5816967; doi:10.7717/peerj.4379)
Supplement: Table S3 [file peerj-06-4379-s003.docx]

Table S3 List of SPS proteins used for the construction of the phylogenetic tree.

| **Species** | **Sequence name** | **Accession number** |
| --- | --- | --- |
| *Saccharum officinarum* | SofSPS1 | ADL70859 |
|  | SofSPS2 | AEO46461 |
|  | SofSPS3 | ADM63847 |
| *Actinidia Chinensis* | AchSPS1 | AAL86360 |
| *Arabidopsis thaliana* | AtSPS1 | NP_197528 |
|  | AtSPS2 | NP_196672 |
|  | AtSPS3 | NP_171984 |
|  | AtSPS4 | NP_192750 |
| *Bambusa oldhamii* | BoSPS | AAR16190 |
| *Beta vulgaris* | BvSPS1 | CAA57500 |
| *Coffea canephora* | CcSPS | ABI17900 |
| *Cucumis melo* | CmSPS | ABF47344 |
| *Ipomoea batatas* | IbSPS | AAL34531 |
| *Lolium perenne* | LpSPS | BAE80113 |
| *Medicago sativa* | MsSPS | AAK09427 |
| *Triticum aestivum* | TaSPS1 | AAQ14552 |
|  | TaSPS9 | AAQ10452 |
| *Viscum album subsp. album* | VaSPS | AAP94624 |
| *Nicotiana tabacum* | NtSPS1 | AAF06792 |
|  | NtSPS2 | ABA64521 |
|  | NtSPS3 | ABA64520 |
| *Oncidium Goldiana* | OgSPS | AAN11294 |
| *Solanum tuberosum* | StSPS | CAA51872 |
| *Oryza sativa* | OsSPS1 | Q0JGK4 |
|  | OsSPS2 | ABA92286 |
|  | OsSPS3 | AAC49379 |
|  | OsSPS4 | BAC92378 |
|  | OsSPS5 | BAD37372 |
|  | OsSPS7 | BAD25068 |
| *Physcomitrella patens* | PpaSPS1 | AAZ85399 |
|  | PpaSPS2 | AAZ85400 |
| *Prunus persica* | PpeSPS1 | ABV32551 |
|  | PpeSPS2 | ABV32550 |
| *Pyrus pyrifolia* | PpySPS | BAG30918 |
| *Sorghum bicolor* | SbSPS | ACX94229 |
| *Solanum lycopersicum* | SlSPS1 | AAU29197 |
|  | SlSPS2 | AFD64638 |
|  | SlSPS3 | BAB18136 |
| *Spinacia oleracea* | SolSPS | AAA20092 |
| *Vicia faba var. minor* | VfSPS | CAA91217 |

| **Species name** | **Sequence name** | **Accession number** |
| --- | --- | --- |
| *Xerophyta humilis* | XhSPS1 | ADG01611 |
|  | XhSPS2 | ADG01610 |
| *Zea mays* | ZmSPS1 | AAA33513 |
| *Citrus unshiu* | CuSPS | BAA23213 |
| *Mangifera indica* | MiSPS | BAM68538 |
| *Populus trichocarpa* | PtSPS | XP_002324874 |
| *Lycium barbarum* | LbSPS | AKA43893 |
| *Vitis vinifera* | VvSPS1 | NP_001267857 |
| *Dimocarpus longan* | DlSPS | AJW82919 |
| *Litchi chinensis* | LcSPS1 | AFP23360 |
