# Supplementary material for: Identification and expression profile analysis of the sucrose phosphate synthase gene family in Litchi chinensis Sonn
Source: PeerJ. 2018 Feb 15;6:e4379. doi: 10.7717/peerj.4379 (PMC5816967; doi:10.7717/peerj.4379)
Supplement: Supplemental Information 1 [file peerj-06-4379-s004.docx]

**CDS sequence**

>*LcSPS1*

ATGGCAGGAAACGACTGGATAAACAGTTACCTGGAAGCGATACTGGATGTCGGACCAGGTCTCGACGACGCCAAATCCTCTCTTCTTCTCCGAGAGAGAGGCAGGTTCAGTCCTACTCGTTACTTCGTCGAAGAAGTCATCACCGGATTCGATGAAACCGATCTCCACCGATCCTGGATTCGAGCTCAAGCGACGAGGAGTCCGCAGGAGAGGAATACAAGGTTGGAGAATATGTGTTGGAGGATTTGGAACTTGGCTCGTCAGAAAAAGCAGCTTGAGGGGGAGGCGGTCCAAAGGATGGCTAAGCGTCGTCAAGAACGTGAAAGAGCTCGCAGGGAAGCAACTGCTGATATGTCTGAAGACTTATCAGAAGGCGAGAAAGGAGATACAGTTAGTGATTTATCAGCTCATGGTGATCATACCAGAGCCAGACTACCTAGAATAAATTCTGTCGATGTAATGGAGACATGGGTTAGTCAACAGAAGGGAAAGAAGCTATACATTGTATTAATAAGTCTTCATGGTCTCATACGAGGAGAAAATATGGAGCTTGGTCGTGATTCTGATACTGGTGGTCAGGTCAAATATGTTGTAGAACTTGCAAGAGCTTTGGGCTCAATGCCAGGAGTTTATCGGGTTGATTTGCTGACTAGACAGGTATCAGCACCAGATGTGGATTGGAGTTATGGTGAACCCACAGAGATGCTGACTCCAAGAAACTCAGAAGATTGCATGGATGAGATGGGGGAAAGCAGTGGCGCTTACATAATTCGTATACCATTTGGTCCGAAAGATAAATATATTCCCAAAGAACTGTTGTGGCCGCACATTCCTGAGTTCGTTGATGGTGCATTTAACCACATAATACAGATGTCCAGTGTTCTTGGGGAGCAAGTTGGTGGTGGGAAACCTGTCTGGCCCATTGCTATCCATGGGCATTATGCAGATGCAGGTGACGCTGCTGCTCTTCTATCTGGTGCTCTAAATGTACCCATGCTTTTTACTGGCCACTCACTTGGCCGAGATAAGTTAGAACAGCTATTGAAACAAGGCCGGTTATCAAGGGATGAAATAAACAAAACGTACAAAATAATGCGTAGAATTGAGGCTGAAGAATTGTCTCTTGATGCCTCTGAGATTGTGATAACTAGCACTAGACAGGAGATTGAGGAGCAATGGCGTTTATATGATGGTTTTGACCCAGTACTGGAACGTAAACTACGAGCTAGGATCAGGCGTAATGTGAGCTGTTATGGCAGGTTCATGCCTCGCATGGCAATAATTCCTCCTGGAATGGAGTTCCATCATATTGTTCCAGTAGATGGTGACATGGATGGTGAAACAGAAGGAAACGAAGACCACCCCTCTTCTCCGGATCCACACATCTGGTCTGAGATAATGCGCTTCTTTACCAACCCACGCAAGCCTATGATACTTGCACTTGCTAGGCCAGATCCAAAAAAGAATATCACAACTTTGGTTAAGGCATTTGGAGAATGCCGTCCACTAAGAGAGCTTGCTAACCTTACTCTAATTATGGGTAACCGAGATGGAATTGATGAAATGTCAAGCACAAATGCATCCGTCCTTCTCTCAGTCATTAAGCTTATTGACAAATATGATCTGTACGGGCAAGTGGCATACCCTAAACATCACAAACAATCTGATGTTCCCGACATATATCGTCTTGCGGCAAAGACAAAGGGTGTCTTTATTAATCCAGCTTTCATTGAGCCATTCGGGCTTACTTTAATTGAGGCAGCAGCTCATGGTTTGCCTATGGTTGCCACAAAAAATGGAGGTCCTGTCGATATACATCGGGTACTTGACAATGGTCTTCTTATTGATCCCCATGATCAACAGTCAATTGCTGATGCTCTTCTGAAGCTTGTTGCTGATAAGCAGCTTTGGGCAAAGTGTCGACAAAATGGATTAAAGAACATCCACCTATTTTCCTGGCCAGAGCATTGTAAAAGTTACCTAACTCGGATAGCCAGTTGCAAGCCAAGGTATCCACAGTGGCTAAAAGATGATGATGGAGGTGAAACATCAGAATCAGATTCACCGGGCGATTCCTTGAGAGATATACATGATATATCTTTGAATTTGAAATTTTCATTGGATGGAGAAAAGAATGGATCTAGTGGAAACGATAATTCTTTAGAGTCTGAAGGAAATGTTTCTGAGAGGAGGAGTAAAATAGAGAATGCTGTTTTGGCCTGGTCAAAGGGTGTTTTAAAGGAGCCACGAAAGGCTGGGACCACCGATAAAGCTGACCAAAACACCAGTGCTGGTAAATTTCCTGCAGTGAGGAGGCGAAAACATATCTTTGTCATTTCTGTGGATTACGATACCACTGCAGGTCTTCTTGAAACTATTAGAAAGATATTTGAGGCTGTGGGTAAGGAAAAGACTGAAGGCTCTATAGGATTTATATTGTCAACATCTCTGACCATATCTGAGATGCACTCGTTCCTGGTCTTGGGGGGCTTCAGCCCTAGCGATTTTGATGCATTTATTTGTAATAGTGGTAGTGATCTATACTATTCAACGCTTAATCCTGAGGATGGTCCTTTTGTGCTCGACTTTTATTACCATTCTCACATTGAGTACCGCTGGGGCGGGGAAGGTTTAAGGAAGACTTTGATTCGTTGGGTAGCTTCAGTTGCTGATAAGAAGGCTGAGAATGAGGAAAAAATTGTCACAGCAGCTGAACAACTTTCAACCAACTATTGTTATGCTTTTACCGTGCAAAAGCCTGGAATGGTTACCCCAGTTAAGGAGCTCAGAAAATTGCTGAGAATCCAAGCACTTCGTTGTCATGTAATTTATTGTCAAAATGGCACCAGGATAAATGTGATTCCAGTTTTGGCATCCCGTTCCCAAGCCCTCAGGTATCTATATCTCCGATGGGGCGTGGAGTTGTCAAAGATGGTAGTTTTTGTTGGAGAATGTGGGGACACAGATTATGAAGGACTGCTTGGTGGCCTGCACAAAAGCATAATATTGAAGGGAGCTTGTAGCAGTGCGAGCAATCAAGTCCATGCTAACCGAAGTTACCCTCTCTCAGATGTCATGCCATGTGACAGCCCAAATATTGTTGAGACACCTGAAGATTTCACTAGCTCTGATGTCCGCAATTCGCTGGAGAGTTTAGGAATTCTTAAGGCCTAG

>*LcSPS2*

ATGGCGGGAAACGACTGGGTTAACAGTTACTTGGAGGCGATCCTGGACGTTGATCCGGGAATCGACAACACGAAATCGTCGCTTTTGCTTAGAGAGAGAGGACACTTCAGTCCTACTCGCTACTTCGTGGAACAGGTCATCACCGGCTTCGATGAAACCGATCTCCACCGCTCCTGGGTTCGAGCTGCGGCGACGAGGAGTCCACAGGAGAGGAACACTAGATTGGAGAACATGTGTTGGAGGATCTGGAATCTCGCTCGCAAGAAGAAGCAGCTTGAGGCAGAGGAAGCTCACCGGTTTACTAAACGTCGGCTTGAACGTGAAAGAGGGCGCAGAGAGGCAACGGCAGATATGTCAGAAGATTTATCAGAGGGAGAGAAAGGAGACATGCCTGGTGACCTTTCGGCTCATGGTGGTGATAGCACTAGAGGAAGGATGCCTAGAATCAGTTCTGTTGATGTGATGGACAATTGGGTGAATCAATACAAGGGGAAGAAACTCTACATTGTGTTGATAAGTCTTCATGGCCTGATACGTGGAGAAAACATGGAGCTTGGTCGTGATTCTGATACAGGTGGACAGGTCAAGTATGTCGTAGAACTTGCAAGGGCCTTGGGCACAATGCCAGGAGTTTATCGGGTTGACTTGCTGACCAGACAAGTATCGGCTCCTGACGTAGACTGGAGCTATGCTGAACCAACAGAGATGTTGAATCTTAGAAGCACTTACAATTCAAATCAAGAGCTCGGGGAGAGCAGCGGTGCATATATCATCCGTATACCATTCGGACCAAAAAATAAATATATACCTAAAGAACAGCTTTGGCCCCACATTCCAGAATTTGTTGACGCTGCACTTAGTCACATCATACAGATGTCAAAAGTTCTGGGTGAGCAAATTGGTGGTGGACAACTAGTCTGGCCGGTTGCAATTCATGGACATTATGCTGATGCAGGTGACTCTGCTGCTCTTCTGTCTGGAGCTCTGAATGTGCCAATGGTTTTGACCGGTCACTCACTTGGACGAGATAAGCTTGAACAACTTTTGAAACAAGGACGCCAATCAAGAGAAGAAATAAATTCAGCATACAAAATAATGCGGCGGATAGAAGCCGAGGAGTTATCTCTTGATGCCTCTGAAATTGTTATAACTAGCACTAGACAGGAGATAGAAGAGCAATGGCGCCTTTATGATGGTTTTGATCCAGTGCTGGAGCGCAAACTGAGAGCTAGGATCAAAAGAGGTGTAAGCTGTCATGGCAGGTTTATGCCTCGTATGGTTGTAATTCCTCCTGGAATGGAGTTCCATCATATTGTGCCGCATGACGGTGATATGGATGGGGAAGTGGAAAAAAATGAAGACAATCCTGCTTCTCCTGATCCACCGATTTGGTCTGAGATAATGCGTTTCTTTTCCAACCCACGCAAGCCTATGATACTTGCTCTTGCCCGGCCAGACCCCAAAAAGAATATTACAACCTTGGTTAAAGCATTTGGAGAATGCCGTCCCCTAAAGGAGCTTGCTAACCTTACACTAATTATGGGAAATCGTGATGATATTGAGGAAATGTCTGGCACAAATGCATCTGTGCTTCTTTCAATTCTTAAGTTAATTGACAAGTATGATCTTTATGGTCAAGTGGCCTATCCGAAACACCACAAGCAATCTGATGTTCCAGAGATCTATCGTTTGGCAGCAAAAACAAAGGGTGTTTTCATCAATCCAGCTTTCATCGAACCTTTTGGGCTCACTTTAATTGAGGCTGCAGCTTATGGCTTGCCTATGGTTGCCACAAAAAATGGAGGTCCTGTTGACATCCATCGGGTTCTCGATAATGGTCTACTTGTGGACCCTCATGATCAGCAATCTATAGCTGACGCTCTTCTGAAGCTTGTTTCAGATAAGCAACTTTGGGCAAGATGCAGGCAGAATGGATTGAAAAACATTCATCTGTTTTCATGGCCTGAGCACTGTAGAAACTACTTATTTCGGATAGCCAGTTGCAGGCCAAGGCAGCCTCAGTGGCAGAGAAGTGATGAGGGACCTGACAATAGAGAAGCTGATTCACCTAGTGATTCCTTGAGGGACATACATGATTTATCTTTGAACTTGAAGCTTTCGCTCGATGGTGACAAAAATGAAGGTGGTAGTACTCTTGACAGTGCTTTGGATTCTGAAGAAAATCCTGTTAATGAGAAGAAAAAGTTGGAGAATGCCATTTTGACGTTGTCCAAGGGTGCAATTGGAGGAGCACAAAAGGCAGACCAGAACATTGGTGCTGGTAAATTCCCAGCGTTCAGGGGGAGGAAGTATATTTTTATCATAGCTGTGGATTGTGATACAACCTCAGATGTTCTCAAAATTATTAAAATGGTTATTGAGACAGCAAAGAAGGAAAAGTTTGCAGGGTCTATCGGGTTCATACTGTCGACAGCATTGACCATTGCCGAGGTCAATTCTCTTATAGTAGCAGGAGGTTTGACCCCATCAGATTTTGATGCTTACATCTGCAATAGTGGTAGTGAGCTCTACTATCCGTCTTCAAGCACTGAGGATGTTCCTGGGCTTCCCTTCGTAGTGGATCTAGATTACCATTCTCATATTGAATACCGATGGGGTGGAGAAGGTTTAAGGAAGACCTTGGTTCGTTGGGCTGCTTCTGTCAATGATAAAAAGGCAGAAGCAGGAAAAATTGTTGAGGAGGATGAATCAGGATCAACTTCGCATTGCTATGCATTCAAAGTCTCAAATCCAACAATGGTGCCACCGTTTAAGGAACTTAGAAAACTAATGAGAATCCAGGCTCTTCGGTGCCATGTTCTTTATTGTCAAAATGGTACCAAGCTTCACGTGATTCCTGTATTGGCTTCTCGATCCCAGGCCCTCAGGTACCTACATGTCCGGTGGGGCATAGATTTATCGAATATGGTGGTCTTTACAGGAGAATGTGGGGACACAGACTATGAAGGCTTGTTTGGTGGAGTCCATAAAACTGTGATTCTTAAGGGAGTTGGTATTGAAGCTCGAAAGCTTCATACTAATAGAAACTACCCGTTAGAGGACGTTATACCGTTCAACAATCCCAATATTATTCAAACCAAGGCTTTCGACAGCAACAGCATAAAATCATCATTGGAAAAACTCGGGGTTCTTTAA

>*LcSPS3*

ATGGCTGGCAACGAGTGGATAAATGGATACTTGGAGGCCATACTGGACAGTGGTGCTGGCGCGGCGGCCATAGAGGACCAGAAGCCTACTCCCGTTAACTTGACGACGGACAGGGGTCATTTCAACCCCACCAAGTATTTCGTGGAGGAGGTGGTTACTGGCGTCGATGAGACTGACCTTTATCGCACTTGGATCAAGGTGGTCGCCACCCGCAACATCCGCGAGCGTAGCTCCAGGCTCGAGAACATGTGCTGGCGCATTTGGCACCTCACCCGCAAGAAGAAACAGCTGGAATGGGAGGAAGTACAACGCTTGGCAAACAGGAGATGGGAGCGGGAGCAGGGGCGCAGGGACGTAACTGAAGACATGTCTGAAGACCTGTCAGAAGGTGAAAAGGGAGATGCATTGGCAGAGCTGGTGCAATGTGAGACTCCAAGGAAAAGTTTCCAACGACAACACTCAAACTTAGAAGTGTGGTCGGACGATAAGAAAGAGAAAAAACTCTACATTGTTCTTATCAGTTTGCATGGTTTGGTCCGGGGAGATAACATGGAGCTTGGTCGGGATTCTGACACTGGTGGACAGGTCAAATATGTGGTAGAGCTTTCCCGTGCACTTGCAAGGATGCCGGGCGTGTATAGGGTAGATCTCTTTACTCGCCAAGTCTCTTCTCCAGAAGTTGATTGGAGTTATGGGGAGCCAACAGAAATGCTGACGTCTGGTGCCGAAGATGCCGATGGCAATGAAGTGGGCGAGAGCAGTGGGGCATATGTTATTAGGATTCCATTTGGTCCGCGTGACAAGTACCTCCGGAAAGAATTACTGTGGCCCTATATTCAGGAGTTCGTAGATGGAGCGCTTGCCCATATTCTAAATATGTCAAAGGTTTTAGGTGAACAAATTGGCAGGGGCCATCCTGTCTGGCCATATGTGATTCATGGCCACTACGCGGATGCAGGGGATAGTGCTGCTCTTCTTTCTGGTGCTTTGAATGTCCCAATGGTTTTGACTGGACATTCACTAGGCAGAAACAAGCTCGAACAACTTCTTAAGCAGGGACGGCAGTCGACGGAGGATATCAATTCAACATATAAGATTATGCGAAGGATAGAAGCAGAGGAGCTTTCCCTTGATGCTGCTGAACTTGTTATCACGAGTACTAAACAGGAGATTGAAGAGCAATGGGGACTTTATGATGGGTTTGACGTGAAGCTTGAGAAAGTTTTACGTGCTCGTGCTAGACGTGGGGTCAATTGTCATGGCCGATACATGCCCAGGATGGTGGTTATTCCCCCTGGCATGGACTTCAGCAATGTTGTGCAAGAAGATGCCCCTGAGGCTGAAGGGGAACTTGCAGCTCTTACTGGTAGCAATGATGGATCTTCTCCAAAAGCAGTTCCAGCAATATGGTCCGAAGTGATGCGGTTCCTTACAAATCCCCACAAACCAATGATCTTAGCTTTATCAAGACCTGACCCAAAGAAGAACATGACAACACTTTTGAAGGCCTTTGGAGAGTGCCGTCCCTTAAGAGAGCTTGCTAATCTTACACTGATAATGGGGAATAGGGATGATATAGAGGAGATGTCTGGAGGAAATGCTAGTGTGCTTACAACAGTATTGAAACTGATTGATAAGTATGACCTCTATGGGCAAGTTGCCTATCCGAAGCATCACAAGCAATCTGATGTTCCAGAAATATACCGACTTGCAGCTAAGACAAAGGGAGTCTTCATAAATCCAGCACTGGTTGAACCATTTGGGCTTACATTGATTGAGGCAGCAGCTCATGGGCTTCCAATGGTGGCTACTAAAAATGGTGGACCAGTTGACATCCATCGGGCTCTGAACAATGGCCTGCTTGTGGATCCCCATGATCAGCAAGCCATTGCTGATGCACTGCTTAAGTTGGTGTCAGAGAAGAACTTATGGCATGATTGCAGAAAGAATGGTTGGAAAAACATACACCTTTTCTCTTGGCCTGAGCACTGTCGCACTTACTTAACAAGGGTAGCAGCTTGCCGAATGAGGCACCCACAGTGGCAAACTGATACTCCAGGGGACGAGATGGCTGCTGATGAATCGTCTCTTAATGACTCACTGAAGGATGTTCAGGATATGTCACTCAGGCTCTCAGTTGATGGAGACAAACCATCACTGAATGAATCTCTCGACTATACAGCTGCCGCAACGGGTGATCCCGTGCAGGACCAAGTGAAACGAGTTCTAAGCAAGATCAAGCGACCTGAATCCCCTCCAAAGGATAAGGAAGGTGGGAAGAAACTGCTTGAGAATGTGGTGAGCAAGTATCCCATGTTGAGGCGGCGCCGTAGATTGATTGTTATTGCTCTTGACTGCTATGATAGCACGGGTGCTCCTGACAAGAAGATGATAAAAATAGTGCAAGATATATTAAAAGCTGTTCGTTCGGACACTCAAACAGCAAGATTATCCGGATTTGCTCTGTCAACAGCAATGCCGCTGTCAGAAACAACAAAGTTCTTAAGTTCAGCAGAAATTCAAGTAAATGAGTTTGATGCTCTGATTTGCAGCAGTGGAGGTGAAGTGTATTATCCAGGTACTTACACGGAAGAGGATGGAAAGCTTTTCCCGGATCCAGACTATGCATCACATATTGACTATCGATGGGGTATTGATGGTCTAAAGAAAACCATTTGGAAGCTGATGAATACAAGTGAAGGTGGAGAGAATTCTGTTCAATCTGGCAGCCCCATTCAGGAGGATGAGAAATCGAGCAATGCTCATTGCATTTCATACCTGATTAAAGATCCCAGTAAGGTAAGACGTGTTGATGATTTGAGGCAGAAGCTCAGGATGCGTGGTCTCCGTTGCCATCCTATGTATTGCAGGAACTCAACGAGAATGCAAATCGTTCCTCTTCTAGCCTCTCGAGCACAAGCACTCAGGTATCTTTTTGTTCGATGGAGATTGAATGTTGCAAACATGTATGTGGTTCTCGGTGAAAGCGGTGATACAGATTATGAGGAGTTGATATCTGGAACTCATAAGACTTTAATCGTGAAAGGAGCGGTGCAGAAGGGTTCTGAAGAATTGCTCAGAGTTACTGATCTAAGAGAGGACATTGTTCCTAGTGAGAGCCCTCTGATTGCACACATAAATGCGGAGGCCAAAGTCGATGAAATTGCCAATGTTTTAAAGCAAGTCTCCAAAGCTTCTGTAGGAATGTGA

>*LcSPS4*

ATGGCAGGAAATGAGTGGATAAATGGGTACTTGGAAGCAATTTTGGATGTAGGAAGTAGCACAAGGAAAAAATTTGATGGGAAGTTGAAGCTCTCCAAGTTTGAAGAGTCCAAAGCCACTAAAGAAAAAGGAAAGTTGTTTAGTCCCACTAAGTACTTCGTTGAAGAAGTTATTAATAGCGTTGATGAATCTGATCTCCATAGAACTTGGGTCAAGGTGATAGCAACAAGAAACTCTCGTGAACGCAGTAACCGGCTCGAGAATATGTGCTGGCGCATTTGGCATCTCACCCGTAAAAAGAGACAGATTGCATGGGAGGATGCACAAAGGCTTGCAAAGAGACGACTGGAGAGGGAGCAAGGTCGTAATGATGCTGCCGAAGATCTTTCCGAGCTCTCTGAAGGCGAGAAGGAGAAGGGCGATACCAATGTCTCCGAATCTGTCAAGGACATACCGAGAATCAACTCTGATATGCAAATTTGGTCCGACGATCATAACTCCGGCAGCCTTTACATTGTCTTGATCAGTTTGCATGGATTGGTGCGTGGAGAAAATATGGAGCTTGGAAGAGATTCTGATACTGGTGGACAGGTGAAATATGTTGTTGAACTTGCTCGAGCCTTGGCAAACACAAAGGGTGTGTATCGTGTGGATCTCCTCACTAGACAAATTGCCTCACCCGAGGTCGACTACAGCTACGGTGAGCCCATTGAGATGCTTTCCTGCCCCTCCGACGGCAGCGATAGCTGTGGAGCCTACATCATCCGAATCCCATGTGGTCCTCGTCACAAGTACATACCAAAAGAGTCACTATGGCCTCATATTCCTGAATTTGTTGATGGAGCTTTGAGTCACATTGTGAACATGGCAAGGGTACTAGGAGAGCAAGTCAATGGAGGAAAACCAACATGGCCCTATGTGATTCACGGGCACTACGCAGATGCTGGAGAGGTGGCGGCACACCTCTCTGGTGCCTTGAATGTGCCAATGGTACTAACGGGGCACTCATTGGGGCGGAACAAGTTCGAGCAGTTGCTCAAACAAGGGAGGCTTTCTAAGGAGGACATAAATGCAACCTACAAGATCATGAGGAGGATTGAAGCTGAAGAGTTGGGGCTGGATTCTGCTGAAATGGTGGTGACTAGCACAAGGCAGGAGATAGAAGAACAATGGGGGTTGTATGATGGGTTTGATATCAAGTTGGAGAGGAAGCTGAGGGTCAGGAGGCAGCGTGGAGTGAGCTGCCTTGGACGTTACATGCCAAGGATGGTGGTTATACCACCAGGAATGGACTTTAGCTACGTTAATACAAAAGATTCATTGGAGGGTGATCTGAAATCATTGATTGGCTCTGATAGAACTCAAAGCAAAAGACATCTGCCTCCTATTTGGTCTGAGATTATGAGATTTTTCACAAATCCACACAAGCCAACTATACTAGCCTTGTCCCGTCCTGACCCGAAAAAGAATGTCACCACATTGCTCAAGGCTTTTGGGGAGTGCCAGCCACTCCGAGAGCTAGCCAACTTGACACTAATACTAGGAAACAGAGATGATATTGAAGAGATGTCAAACAGCAGCTCAGTTGTTCTTACTACAGTACTCAAGCTCATTGACAAGTACGACTTGTACGGTCAGGTGGCCTATCCCAAGCATCATAAGCAATCTGAAGTACCTGAAATTTATCGTCTGGCTGCAAAAACAAAGGGAGTTTTCATCAATCCAGCTCTGGTGGAACCATTTGGTCTCACACTCATTGAGGCAGCTGCATATGGTTTACCAATTGTCACCACCAAAAATGGAGGACCTGTGGACATTGTGAAGGCACTTAACAATGGCCTCTTAATTGATCCCCATGATCATAAAGCCATTGCAGATGCCCTATTAAAGCTGGTTGCGGACAAGAACATGTGGTCTGAATGTAGGAAAAATGGCCTCAAGTATATCCACCGCTTTTCATGGACAGAACACTGCCGTAACTACCTCTCCCATGTAGAACACTGCAGGAACCGCCACCCAACAGCCCGTCTTGAGATCATGAAGGTTCCTGAAGAACCAATGAGTGACTCCTTAAAGGATGTGGAAGACCTCTCTTTGAGATTCTCTGTGGATGGAGACTTCAAGCTTAATGGGGAGCTTGATGCAGCAACCAGGCAGAAGAAACTCATTGAAGCCATCACTCAACAGGCTTGTTTCAATGGGAATACAAGTGCCACTCACAGTCCCGGTAGAAGACAAGTTCTATTTGTAATAGCTGTGGATTGCTATGACAGCAATGGTGACACCACGGAGAACTTCCAAGCAGTTACCAAGAATGTAATGAAAGGTGCAGAACTGAGTCTAGGCTTGGGGAAAGTAGGCTTTCTATTGGTGACAGGTTCAAGTTTACGAGAGACCATGGAAGCACTAAGAAGTTGCCCAGTAAACATAGAAGATTTTGATGCATTGATTTGCAATAGTGGAAGTGAAATCTACTATCCATGGCGAGACATGGTGGCTGATTCAGACTTCGAGGTTCATGTGGAGTACAGATGGCCTGGTGAGAATGTGAGATCAATGGTGCCTAGGCTTGCTAGAGTAGAAGATGGAGCTGAGGATGACATCTCTGAGTATGCGATTGCCTGCAGTGCCAGATGCTATTCTTATAACGTAAAACCAGCAGCCAAGGCTCGAAGAGTAGAAGATCTTCATCAAAGGCTTCGAATGAGAGGCATCCGATGCAACATTATCTTCACACGAGCAGCATCAAGGTTGAATGTAGTGCCATTGTTTGCGTCAAGAATACAAGCTCTAAGGTATCTATCAGTTAGGTGGGGAATAGATCTTTCAAAAATGGTTATGTTTGTGGGCGTAAGAGGAGATACCGACTACGAAAGCCTGATAGCTGGCCTCCATAAGACAATAATTCTAAGAGATGCTGTGGTGTGTGGCAGTGAGAAGCTTGTTCACTGTGAAGATGGTTTCAAAAGCGAAAATGAAGTCCCAGAAGGCAGCAGCAACGTCACCTATGTAGAGGAAGGTTTTGAAGCTCAGAATATCTCTGCAGCTATAAAGGTTCTTCAGATCAAGTGA

**Deduced animo acid sequence**

>*LcSPS1*

MAGNDWINSYLEAILDVGPGLDDAKSSLLLRERGRFSPTRYFVEEVITGFDETDLHRSWIRAQATRSPQERNTRLENMCWRIWNLARQKKQLEGEAVQRMAKRRQERERARREATADMSEDLSEGEKGDTVSDLSAHGDHTRARLPRINSVDVMETWVSQQKGKKLYIVLISLHGLIRGENMELGRDSDTGGQVKYVVELARALGSMPGVYRVDLLTRQVSAPDVDWSYGEPTEMLTPRNSEDCMDEMGESSGAYIIRIPFGPKDKYIPKELLWPHIPEFVDGAFNHIIQMSSVLGEQVGGGKPVWPIAIHGHYADAGDAAALLSGALNVPMLFTGHSLGRDKLEQLLKQGRLSRDEINKTYKIMRRIEAEELSLDASEIVITSTRQEIEEQWRLYDGFDPVLERKLRARIRRNVSCYGRFMPRMAIIPPGMEFHHIVPVDGDMDGETEGNEDHPSSPDPHIWSEIMRFFTNPRKPMILALARPDPKKNITTLVKAFGECRPLRELANLTLIMGNRDGIDEMSSTNASVLLSVIKLIDKYDLYGQVAYPKHHKQSDVPDIYRLAAKTKGVFINPAFIEPFGLTLIEAAAHGLPMVATKNGGPVDIHRVLDNGLLIDPHDQQSIADALLKLVADKQLWAKCRQNGLKNIHLFSWPEHCKSYLTRIASCKPRYPQWLKDDDGGETSESDSPGDSLRDIHDISLNLKFSLDGEKNGSSGNDNSLESEGNVSERRSKIENAVLAWSKGVLKEPRKAGTTDKADQNTSAGKFPAVRRRKHIFVISVDYDTTAGLLETIRKIFEAVGKEKTEGSIGFILSTSLTISEMHSFLVLGGFSPSDFDAFICNSGSDLYYSTLNPEDGPFVLDFYYHSHIEYRWGGEGLRKTLIRWVASVADKKAENEEKIVTAAEQLSTNYCYAFTVQKPGMVTPVKELRKLLRIQALRCHVIYCQNGTRINVIPVLASRSQALRYLYLRWGVELSKMVVFVGECGDTDYEGLLGGLHKSIILKGACSSASNQVHANRSYPLSDVMPCDSPNIVETPEDFTSSDVRNSLESLGILKA

>*LcSPS2*

MAGNDWVNSYLEAILDVDPGIDNTKSSLLLRERGHFSPTRYFVEQVITGFDETDLHRSWVRAAATRSPQERNTRLENMCWRIWNLARKKKQLEAEEAHRFTKRRLERERGRREATADMSEDLSEGEKGDMPGDLSAHGGDSTRGRMPRISSVDVMDNWVNQYKGKKLYIVLISLHGLIRGENMELGRDSDTGGQVKYVVELARALGTMPGVYRVDLLTRQVSAPDVDWSYAEPTEMLNLRSTYNSNQELGESSGAYIIRIPFGPKNKYIPKEQLWPHIPEFVDAALSHIIQMSKVLGEQIGGGQLVWPVAIHGHYADAGDSAALLSGALNVPMVLTGHSLGRDKLEQLLKQGRQSREEINSAYKIMRRIEAEELSLDASEIVITSTRQEIEEQWRLYDGFDPVLERKLRARIKRGVSCHGRFMPRMVVIPPGMEFHHIVPHDGDMDGEVEKNEDNPASPDPPIWSEIMRFFSNPRKPMILALARPDPKKNITTLVKAFGECRPLKELANLTLIMGNRDDIEEMSGTNASVLLSILKLIDKYDLYGQVAYPKHHKQSDVPEIYRLAAKTKGVFINPAFIEPFGLTLIEAAAYGLPMVATKNGGPVDIHRVLDNGLLVDPHDQQSIADALLKLVSDKQLWARCRQNGLKNIHLFSWPEHCRNYLFRIASCRPRQPQWQRSDEGPDNREADSPSDSLRDIHDLSLNLKLSLDGDKNEGGSTLDSALDSEENPVNEKKKLENAILTLSKGAIGGAQKADQNIGAGKFPAFRGRKYIFIIAVDCDTTSDVLKIIKMVIETAKKEKFAGSIGFILSTALTIAEVNSLIVAGGLTPSDFDAYICNSGSELYYPSSSTEDVPGLPFVVDLDYHSHIEYRWGGEGLRKTLVRWAASVNDKKAEAGKIVEEDESGSTSHCYAFKVSNPTMVPPFKELRKLMRIQALRCHVLYCQNGTKLHVIPVLASRSQALRYLHVRWGIDLSNMVVFTGECGDTDYEGLFGGVHKTVILKGVGIEARKLHTNRNYPLEDVIPFNNPNIIQTKAFDSNSIKSSLEKLGVL

>*LcSPS3*

MAGNEWINGYLEAILDSGAGAAAIEDQKPTPVNLTTDRGHFNPTKYFVEEVVTGVDETDLYRTWIKVVATRNIRERSSRLENMCWRIWHLTRKKKQLEWEEVQRLANRRWEREQGRRDVTEDMSEDLSEGEKGDALAELVQCETPRKSFQRQHSNLEVWSDDKKEKKLYIVLISLHGLVRGDNMELGRDSDTGGQVKYVVELSRALARMPGVYRVDLFTRQVSSPEVDWSYGEPTEMLTSGAEDADGNEVGESSGAYVIRIPFGPRDKYLRKELLWPYIQEFVDGALAHILNMSKVLGEQIGRGHPVWPYVIHGHYADAGDSAALLSGALNVPMVLTGHSLGRNKLEQLLKQGRQSTEDINSTYKIMRRIEAEELSLDAAELVITSTKQEIEEQWGLYDGFDVKLEKVLRARARRGVNCHGRYMPRMVVIPPGMDFSNVVQEDAPEAEGELAALTGSNDGSSPKAVPAIWSEVMRFLTNPHKPMILALSRPDPKKNMTTLLKAFGECRPLRELANLTLIMGNRDDIEEMSGGNASVLTTVLKLIDKYDLYGQVAYPKHHKQSDVPEIYRLAAKTKGVFINPALVEPFGLTLIEAAAHGLPMVATKNGGPVDIHRALNNGLLVDPHDQQAIADALLKLVSEKNLWHDCRKNGWKNIHLFSWPEHCRTYLTRVAACRMRHPQWQTDTPGDEMAADESSLNDSLKDVQDMSLRLSVDGDKPSLNESLDYTAAATGDPVQDQVKRVLSKIKRPESPPKDKEGGKKLLENVVSKYPMLRRRRRLIVIALDCYDSTGAPDKKMIKIVQDILKAVRSDTQTARLSGFALSTAMPLSETTKFLSSAEIQVNEFDALICSSGGEVYYPGTYTEEDGKLFPDPDYASHIDYRWGIDGLKKTIWKLMNTSEGGENSVQSGSPIQEDEKSSNAHCISYLIKDPSKVRRVDDLRQKLRMRGLRCHPMYCRNSTRMQIVPLLASRAQALRYLFVRWRLNVANMYVVLGESGDTDYEELISGTHKTLIVKGAVQKGSEELLRVTDLREDIVPSESPLIA

HINAEAKVDEIANVLKQVSKASVGM

>*LcSPS4*

MAGNEWINGYLEAILDVGSSTRKKFDGKLKLSKFEESKATKEKGKLFSPTKYFVEEVINSVDESDLHRTWVKVIATRNSRERSNRLENMCWRIWHLTRKKRQIAWEDAQRLAKRRLEREQGRNDAAEDLSELSEGEKEKGDTNVSESVKDIPRINSDMQIWSDDHNSGSLYIVLISLHGLVRGENMELGRDSDTGGQVKYVVELARALANTKGVYRVDLLTRQIASPEVDYSYGEPIEMLSCPSDGSDSCGAYIIRIPCGPRHKYIPKESLWPHIPEFVDGALSHIVNMARVLGEQVNGGKPTWPYVIHGHYADAGEVAAHLSGALNVPMVLTGHSLGRNKFEQLLKQGRLSKEDINATYKIMRRIEAEELGLDSAEMVVTSTRQEIEEQWGLYDGFDIKLERKLRVRRQRGVSCLGRYMPRMVVIPPGMDFSYVNTKDSLEGDLKSLIGSDRTQSKRHLPPIWSEIMRFFTNPHKPTILALSRPDPKKNVTTLLKAFGECQPLRELANLTLILGNRDDIEEMSNSSSVVLTTVLKLIDKYDLYGQVAYPKHHKQSEVPEIYRLAAKTKGVFINPALVEPFGLTLIEAAAYGLPIVTTKNGGPVDIVKALNNGLLIDPHDHKAIADALLKLVADKNMWSECRKNGLKYIHRFSWTEHCRNYLSHVEHCRNRHPTARLEIMKVPEEPMSDSLKDVEDLSLRFSVDGDFKLNGELDAATRQKKLIEAITQQACFNGNTSATHSPGRRQVLFVIAVDCYDSNGDTTENFQAVTKNVMKGAELSLGLGKVGFLLVTGSSLRETMEALRSCPVNIEDFDALICNSGSEIYYPWRDMVADSDFEVHVEYRWPGENVRSMVPRLARVEDGAEDDISEYAIACSARCYSYNVKPAAKARRVEDLHQRLRMRGIRCNIIFTRAASRLNVVPLFASRIQALRYLSVRWGIDLSKMVMFVGVRGDTDYESLIAGLHKTIILRDAVVCGSEKLVHCEDGFKSENEVPEGSSNVT

YVEEGFEAQNISAAIKVLQIK

**Genomic sequence**

>*LcSPS1*

ATGGCAGGAAACGACTGGATAAACAGTTACCTGGAAGCGATACTGGATGTCGGACCAGGTCTCGACGACGCCAAATCCTCTCTTCTTCTCCGAGAGAGAGGGAGGTTCAGTCCAACTCGTTACTTCGTCGAAGAAGTCATCACCGGATTCGATGAAACCGATCTCCACCGATCCTGGATTCGAGTACTGATACACACACACACACGCACGCGCACACACACTTGCATGTACTCTTGTACTTGTAACTTTAACGTCTATCGATATTTATGTGTGATGGTGTGTGAGTGTGTTTCAGGCTCAAGCGACGAGGAGTCCGCAGGAGAGGAATACAAGGTTGGAGAATATGTGTTGGAGGATTTGGAACTTGGCTCGTCAGAAAAAGCAGGTTTTATTATTATTATTATTATTATTTAATTTGTAAGTCTTGTTTTGTTAAAGTTTTATCGTTTTTGTTATCTGATGTTGTATTTAGATTGTGAGGTTTAGTAGTATGATTTGCAGGTGAGTTTTGAAATTTGAATTGATATTATTGGAAGAAAGATGTTTGCTTCTTTTTTTTCTTTCTTTTTTTTTTTAAATATGATTTAATTTATAATTGAAATTAGTACTGTTTACTTATTATTTGGTTGGATTTTTGTATGTAAAAGATAGCATTTGTTTGAAACTGTTATAGTTATATATGATAGTTAGATTTTGGAGTAAGTTATGGCCATGGTTGTGAGAGATTGGTGGGGTGTTTGGTGGATTTTGTGAAAATGTGAAATTTTTATTTGTTTTTTGGTTTCACTTGCTCTCATTGTGTTGATGGACAAATTATGATTCATGATTGTTGTGCAATTACACTGAATCATATCTATGAACCCACAACAATGATAACAGTAACAGACGGTACATTTTTTAGTTATTTTCTGGCTTGTTTTTAGAAGTAGAGAGGGTCGAGGTGTGTTGATGATTTATTGTCTATATTTGTGTAGCTTGAGGGGGAGGCGGTCCAAAGGATGGCTAAGCGTCGTCAAGAACGTGAAAGAGCTCGCAGGGAAGCAACTGCTGATATGTCTGAAGACTTATCAGAAGGTGAGAAAGGAGATACAGTTAGTGATTTATCTGCTCATGGTGATCATACCAGAGCCAGACTACCTAGAATAAATTCTGTCGATGTAATGGAGACATGGGTTAGTCAACAGAAGGGAAAGAAGCTATACATTGTATTAATAAGGCAAGAACTGAACTTTAGTTATCTAGTTTTGTTTGTTTATGTCTACAGGAATGTGAAGTGATGAGTTCGTGTACAGTATTGTCACTATTTGTTTGATGAAACTGTCAATAAACTTGTGTTCCTGTTAATTGATGTAAGCACTGTGATCATTGCTTGAATATAAATTTATTAGTTGTCCGAGGAAACTTAGTGGCTGACAAAACATGTTTCATATGTGCTAAGAACGTCACTTATGATCATTCTGTTATGAATGGTATCTGTTTTTTGTTGAAATTCCCCTTAAATGGAAAACCAAGCCCCTCCAAAACCTCGAATGAGCGGCTTTTGAACCCAAATTCTTTCAATTAAATATGAGAGATCTTACCAGTTGAATCAACAATTTACTTGAATAATTGAAGGAATTGATAAGCACCAAATGATTTCTAGTGGTATATTCTGGGTGGTTTTGTGATTTCACTTGTGAGGCAAGGTACATGGTTGTTCCTCAGTTAATTTACATACTGTAAACATCTAATAATATGTAAGATTCAGCTTTTTATGGTGGCTAGGTATCTTAATAAGATGTTTTTCTTTCATAGATTCAGTTAGTAGCATCTGATTAAAAGGTTTTTGATTTGAATTTTGATATTTAACTGTACTTAACTACTAGTATCTGTGCAGTCTTCATGGTCTCATACGAGGAGAAAATATGGAGCTTGGTCGTGATTCTGATACTGGTGGTCAGGTAATGTTTGGTTGCCAAAGCTTTCTGATGTTGCTATTTTGGTCCCACCTTAGAAAACTTTTACAGGTATTGATTTGGATAACTGACAGATCTGTTGATTTTCTTTCTTTTAGGTCAAATATGTTGTAGAACTTGCAAGAGCTTTGGGCTCAATGCCAGGAGTTTATCGGGTTGATTTGCTGACTAGACAGGTATCAGCACCAGATGTGGATTGGAGTTATGGTGAACCCACAGAGATGCTAACTCCAAGAAACTCAGAAGATTGCATGGATGAGATGGGGGAAAGCAGTGGCGCTTACATAATTCGTATACCATTTGGTCCGAAAGATAAATATATTCCCAAAGAACTGTTGTGGCCGCACATTCCTGAGTTCGTTGATGGTGCATTTAACCACATAATACAGATGTCCAGTGTTCTTGGGGAGCAAGTTGGTGGTGGGAAACCTGTCTGGCCCATTGCTATCCATGGGCATTATGCAGATGCAGGTGACGCTGCTGCTCTTCTATCTGGTGCTCTAAATGTACCCATGCTTTTTACTGGCCACTCACTTGGCCGAGATAAGTTAGAACAGCTATTGAAACAAGGCCGGTTATCAAGGGATGAAATAAACAAAACGTACAAAATAATGCGTAGAATTGAGGCTGAAGAATTGTCTCTTGATGCCTCTGAGATTGTGATAACTAGCACTAGACAGGAGATTGAGGAGCAATGGCGTTTATATGATGGTTTTGACCCAATACTGGAACGTAAACTACGAGCTAGGATCAGGCGTAATGTGAGCTGTTATGGCAGGTTCATGCCTCGCATGGCAGTAAGTACTTTTGACATATGATGAATTGAGCTCAGAAAAAGATTAATTTGTTAAAATAAAGGATGTAATTGCAAAATTTTACTTCATCGTTGATTTCTCTCTCTATTGTAATCCTGCATATCCTGGTAGATTTGTTTCTCTTATATAGTTTACATTTGATGAATGTGTAATTAATGCAGATAATTCCTCCTGGAATGGAGTTCCATCATATTGTTCCAGTAGATGGTGACATGGATGGTGAAACAGAAGGAAACGAAGACCACCCCTCTTCTCCGGATCCACACATCTGGTCTGAGGTATTAATACTATTAGGAATATATTTTAGTCAGTCCTAGGTAGATGGCATCTATATTAAATGATATGGATAATTTTTTGAAGCAAGTACCACATTTTATGCAGTTGTATGCTCGATATACCATTTTTTAAGTTTGTATTTTGCAGATAATGCGCTTCTTTACCAACGCACGCAAGCCTATGATACTTGCACTTGCTAGGCCAGATCCAAAAAAGAATATCACAACTTTGGTTAAGGCATTTGGAGAATGCCGTCCACTAAGAGAGCTTGCTAACCTTGTATGTTCTAAACTAAATGAAACTTTTTGCTGAAGAAATCCTCCTGATAAGCTGAAATGTTATTTTTTTTTTAATTTTTTTTTTCTCCTTTTGTGTTCTCCTCTTTTCCCTGGTGCGGTGCCTTCCAGACTCTAATTATGGGTAACCGAGATGGAATTGATGAAATGTCAAGCACAAATGCATCCGTTCTTCTCTCAGTCATTAAGCTTATTGACAAATATGATCTGTACGGGCAAGTGGCATACCCTAAACATCACAAACAATCTGATGTTCCCGACATATATCGTCTTGCGGCAAAGACAAAGGTAACCATTATGCACCACACTTGTTCTGCTCTTCTTCATGTGTACTATTATATATATATGATCCCATAGAATTTGCCTTAACTACCATCTTCTAGTGTTCAACTTCACGCATCCATTATAACATGTTGGTATTAAATATTGTCTTTTTGTATGGCATTTTGTATGATTTAGTTAGCTATATAGTGATAAAGTTGACTGTTGGGTTGATTGGCTCATCCAGCCTTACTAAGAGCAATTTTTGGCTCTTAAGTCCATTTCTCTTTGAGGCATATTAAGCCCACATTTGTCTTCTGTATCTTCTTGAAGAGGCATATCACTAGGGGTTAATTTGCCTACACCATTATTCTGACAGCCTCTTTTATTATTTATGCTTGCTAAGTAATTGGTTCTTGTACATGCATGTTGTAATCTCTCATATTTTCTTACCCGAAGAATCTACTTGTTCACAGTGGTGTATGTTTATCATGTATCTTATCATTTATCCAACTGTCATGCAGGGTGTCTTTATTAATCCAGCTTTCATTGAGCCATTCGGGCTTACTTTAATTGAGGTACTATTTCTAGTACATATAAAAGTTTTATTTTACTGTTGTTTTCTCAAATTCCTATGTAACAGTGGCTGTTTCACTAAACAGGCAGCAGCTCATGGTTTGCCTATGGTTGCCACAAAAAATGGAGGTCCTGTCGATATACATCGGGTATGCTAACTTTTTTATTGTATATGCTGGTTACGTGATTGTGTCTGAATATATTAGAGCCCAAATGGAAGAGCTTTCACCAAGTTCTCTAAATTTGAATGGAAATGACAGGTACTTGACAATGGTCTTCTTATTGATCCCCATGATCAACAGTCAATTGCTGATGCTCTTCTGAAGCTTGTTGCTGATAAGCAGCTTTGGGCAAAGTGTCGACAAAATGGATTAAAGAACATCCACCTATTTTCCTGGCCAGAGCATTGTAAAAGTTACCTAACTCGGATAGCCAGTTGCAAGCCAAGGTATCCACAGTGGCTAAAAGATGATGATGGAGGTGAAACATCAGAATCAGATTCACCGGGCGATTCCTTGAGAGATATACATGATATATCTTTGAATTTGAAATTTTCATTGGATGGAGAAAAGAATGGATCTAGTGGAAACGATAATTCTTTAGAGTCTGAAGGAAATGTTTCTGAGAGGAGGAGTAAAATAGAGAATGCTGTTTTGGCCTGGTCAAAGGGTGTTTTAAAGGAGCCACGAAAGGCTGGGACGACCGATAAAGCTGACCAAAACACCAGTGCTGGTAAATTTCCTGCAGTGAGGAGGCGAAAACATATCTTTGTCATTTCTGTGGATTACGATACCACTGCAGGTCTTCTTGAAGCTATTAGAAAGATATTTGAGGCTGTGGGTAAGGAAAAGACTGAAGGCTCTATAGGATTTATATTGTCAACATCTCTGACCATATCTGAGATGCACTCGTTCCTGGTCTTGGGGGGCTTCAGCCCTAGCGATTTTGATGCATTTATTTGTAATAGTGGTAGTGATCTATACTATTCAACGCTTAATCCTGAGGATGGTCCTTTTGTGCTCGACTTTTATTACCATTCTCACATTGAGTACCGCTGGGGCGGGGAAGGTTTAAGGAAGACTTTGATTCGTTGGGTAGCTTCAGTTGCTGATAAGAAGGCTGAGAATGAGGAAAAAATTGTCACAGCAGCTGAACAACTTTCAACCAACTATTGTTATGCTTTTACCGTGCAAAAGCCTGGAATGGTAAGTTTTTTTCCTGTTTAACTTGTGAACAAACTTTCCCCATGAGACAACTGCTTTTGTTCTGAAATTGTGGGTAAATTTATTTGTGTTGTTTATGATTTAAAAAAGTTGGGTTTAAATTATTTCCATTGTATCTTATGGGTTTCTGATTTCAGGTTACCCCAATTAAGGAGCTCAGAAAATTGCTGAGAATCCAAGCACTTCGTTGTCATGTAATTTATTGTCAAAATGGCACCAGGATAAATGTGATTCCAGTTTTGGCATCCCGTTCCCAAGCCCTCAGGTAAAATTTTAATTAATTTGGGGATAGTGATTGTTGTCTACACAATTGTCAAGGTTGAATAGGTGCCCTCTGTCATTCATCATTAAAAAAAAGTATAATAAAGTGACAACATGCGAAGGTTAAATGCTTGGTTCATTGCTGACATGACTCCTTAATCAGTGAAGGCAATAGATGTGGGAGTATTAACGCCTATAGAATCTAGTAATTGATCAACTGTTGTTTAAACAAATTATATGTACTTAATGTGAAACAGTATAAATAGTATATTCAATTGGATAAAACCAATTTCATTTAGATATGTAAAACATAAAATAGTAAATATGCATAAAAGCAAATAATTGAATTTTTTATTGGAGCTATGATCTGTTTTCCTCTGTTGCATCGAACTGTCAACTTTGTCCTAGGAATTTTTGTCTGAATTTTGCAGGGACACATGATCAGTGTAGTGGTGGAGTTCTGAACTTTCCAGCTAGTGGCTTTATTATTAGCACAGCACTACACATCATGTAATGGATTTGCTGGGTACAGTGTAATATATCATTTGATGAATTTAGTAGGAAAATAGGGTGATTAGCCAAGCGTCTGAGGGCCTCCTTACAGTACCTCTAGTTATTTTGTCTTGATATTCTTATTCTCAAATGATATGATATAACTATAATACATGTATTTGGCATCATTCTGTAAGTGTAGCATTTTATAAATTTAATGACTTGAAATATATGTTAGGTATCTATATCTCCGATGGGGCGTGGAGTTGTCAAAGATGGTAGTTTTTATTGGAGAATGTGGGGACACAGATTATGAAGCACTGCTTGGTGGCCTGCACAAAAGCATAATATTGAAGGGAGCTTGTAGCAGTGCGAGCAATCAAGTCCATGCTAACCGAAGTTACCCTCTCTCAGATGTCATGCCATGTGACAGCCCAAATATTGTTGAGACATCTGAAGATTTCACTAGCTCTGATGTCCGCAATTCACTGGAGAGTTTAGGAATTCTTAA

GGCCTAG

>*LcSPS2*

ATGGCGGGAAACGACTGGGTTAACAGTTACTTGGAGGCGATCCTGGACGTTGATCCGGGAATCGACAACACGAAATCGTCGCTTTTGCTTAGAGAGAGAGGACACTTCAGTCCTACTCGCTACTTCGTGGAACAGGTCATCACCGGCTTCGATGAAACCGATCTCCACCGCTCCTGGGTTCGAGTACGCGCCACTATCAACCGTCCATTTCGCTTTCCTGTTTTTCGCTTCAAAATTACTTAAAATTGTTGGATTTTGGTGATTGTAGGCTGCGGCGACGAGGAGTCCACAGGAGAGGAACACTAGATTGGAGAACATGTGTTGGAGGATCTGGAATCTCGCTCGCAAGAAGAAGCAGGTTTTGTTTCATGATTCATGTGCAAAATGAAATATTTTTTTGAATTTCTAGTCTATTGATTCACGTGTTTATTAGATATTGCAAGTTGATTTATTTAGAGTTTAAGTGGTTGTTAAAAGGATTGTAACAGTGACACAACATGACCATGTTATCTAGAAAATCTATTGTAGCTTGAACGAGTTTTTGTTTGAATTTATCTTGTTGTGTCTATAGTGTAGGGACACATGCACTTAGATTGGATTTTTCGGATTTTGCTTTAGATGTGCTCTTGTTATGTTTTAGTCTGCATAGTTTCACTGTTTCCATGTTAGTCTAAATAATATGGATGATAAGATTTTTAGTGGTCAAGATTTGTGTTTCAGAAATTTTTAATGAGTTCTAGTTATATGTAGATTTCATTACTCATGAATTTTATCTATTTAATCTATTCGAGTAGCTTGAGGCAGAGGAAGCTCACCGGTTTACTAAACGTCGGCTTGAACGTGAAAGAGGGCGCAGAGAGGCAACGGCAGATATGTCAGAAGATTTATCAGAGGGAGAGAAAGGAGACATGCCTGGTGACCTTTCGGCTCATGGTGGTGATAGCACTAGAGGAAGGATGCCTAGAATCAGTTCTGTTGATGTGATGGACAATTGGGTGAATCAATACAAGGGGAAGAAACTCTACATTGTGTTGATAAGGCATGAACTGCAATTTATTGCGACCTCATGATCGAATAAGGTCATGTGGAGGTTTATTTTGAATGTGCAGTTTTTGTTTATAATTACAAACTCAGATGTGGCAGGGCAGGGTATTTAATTCCAGAGCTTATGAAATTTCTAGTTTTGACGTTTTTAATTTATTTTCTTTAACATGAATTTGTTTTCCAGTCTTCATGGCCTGATACGTGGAGAAAACATGGAGCTTGGTCGTGATTCTGATACAGGTGGACAGGTAACTGTTAGCCATAGCTGTCTTTTGTTTGTCTATTTGTCAAAAAATTTGAAAACCCTGTTAATGTCTTTTACTTTAGTAAGCCTTTGTTGAGACACCATTCTGAAGAACTATTAGTTATGGGAGAGTTTGGAATATGATGTCTAAACAGGTCAAGTATGTCGTAGAACTTGCAAGGGCCTTGGGCACAATGCCAGGAGTTTATCGGGTTGACTTGCTGACCAGACAAGTATCGGCTCCTGACGTAGACTGGAGCTATGCTGAACCAACAGAGATGTTGAATCTTAGAAGCACTTACAATTCAAATCAAGAGCTCGGGGAGAGCAGCGGTGCATATATCATCCGTATACCATTCGGACCAAAAAATAAATATATACCTAAAGAACAGCTTTGGCCCCACATTCCAGAATTTGTTGATGGTGCACTTAGTCACATCATACAGATGTCAAAAGTTCTGGGTGAGCAAATTGGTGGTGGACAACTAGTCTGGCCGGTTGCAATTCATGGACATTATGCTGATGCAGGTGACTCTGCTGCTCTTCTGTCTGGAGCTCTGAATGTGCCAATGGTTTTGACCGGTCACTCACTTGGACGAGATAAGCTTGAACAACTTTTGAAACAAGGACGCCAATCAAGAGAAGAAATAAATTCAGCATACAAAATAATGCGGCGGATAGAAGCCGAGGAGTTATCTCTTGATGCCTCTGAAATTGTTATAACTAGCACTAGACAGGAGATAGAAGAGCAATGGCGCCTTTATGATGGTTTTGATCCAGTGCTGGAGCGCAAACTGAGAGCTAGGATCAAAAGAGGTGTAAGCTGTCATGGCAGGTTTATGCCTCGTATGGTTGTAAGTATTGGCATATTGTCTTTTTTTTTTTCTTTTTAAATAAATAAAATATAAACCTATGATGTTTTTTATACATCTAAAGTTAACTGGTTATATTAATACTTTGCTTTTATTTTTTAATGCAATATTAATTATCCTTATTCATGTCTGACCTTTTTTTAATCATTTATAGGTAATTCCTCCTGGAATGGAGTTCCATCATATTGTGCCGCATGACGGTGATATGGATGGGGAAGTGGAAAAAAATGAAGACAATCCTGCTTCTCCTGATCCACCGATTTGGTCTGAGGTATAATGATTTCTCTTCTGCATTATGAGGGTTGTGATAGCCTGTGTATTGTGGTCCCTTCCACTTAAATATTGTTTTTCTTCAGGCGGGAGCTACCGTAGTATGCTGAGTAACTTTTCCTTGTGCAGTAGAGATTCTGATCACAGTACTGACCTTGGAAGTATTGAATTGCCAGATAATGCGTTTCTTTTCCAACCCACGCAAGCCTATGATACTTGCTCTTGCCCGGCCAGACCCCAAAAAGAATATTACAACCTTGGTTAAAGCATTTGGAGAATGCCGTCCCCTAAAGGAGCTTGCTAACCTTGTAAGTATTCAACATCCACCTGTTGGTTTCTTGATCAAGTGAATTGGTTTCGCATCTTCTTCTTCTTTTTTTTTTTTTTTTTTTTTTTTTTTTTTTGTTTTATTCTGAAAGCAAAAATTGTTTTATGTATAAGTTCTTTTGATAAATTTTTTTATATGTTATTGCTGCAGACACTAATTATGGGAAATCGTGATGATATTGAGGAAATGTCTGGCACAAATGCATCTGTGCTTCTTTCAATTCTTAAGTTAATTGACAAGTATGATCTTTATGGTCAAGTGGCCTATCCGAAACACCACAAGCAATCTGATGTTCCAGAGATCTATCGTTTGGCAGCAAAAACAAAGGTAGAGTTGATGATATAACTTGATGCAAGAACAAGATTAGCTGGCTGCTGTTTCGTGCAGTAAAACATATTAGCACCCGTGGCATATTCTAATATGGACACAACGTTTACAAAGTTTGCATTTTTAATTTTACTTATGCTTTCAGGTGATCTGTATGGTTTCCAAGTAATTACCTAACTATTATTATATTTTAGTGGCTTGATCTTTATATAACACATTTTTTTAATGTTTCTGTCTCATTTAAATTAATTTATTTTGCACAATATGGTTAGGCTCCTTTGTCAGAGGACTGAATGGGGTGGGTTAGTGTATTTGTTGTTCAATTTATATTTCAATTAGATTCATTCTTCTTTTTTTGTTTTTGGTTATTATAGGGTGTTTTCATCAATCCAGCTTTCATCGAACCTTTTGGGCTCACTTTAATTGAGGTTTCTCCTCATCTAATATCTAAAGTCATCTAATGTTATAGAAATTAATTTGTGAACAATCATATCTAAGCTCTTTGCTTGACAGGCTGCAGCTTATGGCTTGCCTATGGTTGCCACAAAAAATGGAGGTCCTGTTGACATCCATCGGGTTTGTCCACTGCTGAATCCTTCTTTAGCTTGTCTATATTTAAGTGTAGCAGTTCATGACATAGTTTATTAGTAATGACTATGAATTTATTACAGGTTCTCGATAATGGTCTACTTGTGGACCCTCATGATCAGCAATCTATAGCTGACGCTCTTCTGAAGCTTGTTTCAGATAAGCAACTTTGGGCAAGATGCAGGCAGAATGGATTGAAAAACATTCATCTGTTTTCATGGCCTGAGCACTGTAGAAACTACTTATTTCGGATAGCCAGTTGCAGGCCAAGGCAGCCTCAGTGGCAGAGAAGTGATGAGGGACCTGACAATAGAGAAGCTGATTCACCTAGTGATTCCTTGAGGGACATACATGATTTATCTTTGAACTTGAAGCTTTCGCTCGATGGTGACAAAAATGAAGGTGGTAGTACTCTTGACAGTGCTTTGGATTCTGAAGAAAATCCTGTTAATGAGAAGAAAAAGTTGGAGAATGCCATTTTGACGTTGTCCAAGGGTGCAATTGGAGGAGCACAAAAGGCAGACCAGAACATTGGTGCTGGTAAATTCCCAGCGTTCAGGGGGAGGAAGTATATTTTTATCATAGCTGTGGATTGTGATACAACCTCAGATGTTCTCAAAATTATTAAAATGGTTATTGAGACAGCAAAGAAGGAAAAGTCTGCAGGGTCTATCGGGTTCATTCTGTCGACAGCATTGACCATCGCCGAGGTCAATTCTCTTATAGTATCAGGAGGTTTGACCCCATCAGATTTTGATGCTTACATCTGCAATAGTGGTAGTGAGCTCTACTATCCGTCTTCAAGCACTGAGGATGTTCCTGGGCTTCCCTTCGTAGTGGATCTAGATTACCATTCTCATATTGAATACCGATGGGGTGGAGAAGGTTTAAGGAAGACCTTGGTTCGTTGGGCTGCTTCTGTCAATGATAAAAAGGCAGAAGCAGGAAAAATTGTTGAGGAGGATGAATCAGGATCAACTTCGCATTGCTATGCATTCAAAGTCTCAAATCCAACAATGGTAATTTTTTTTCCTTTTTTTTGGTTTAATTTTACGCACCCTTAGTTTCTACCACCTTGCTTTTCCTCCTTTGAAGTTATGTTTAATACCTACTCTTTTCTTCACAGGTGCCACCGTTTAAGGAACTTAGAAAACTAATGAGAATCCAGGCTCTTCGGTGCCATGTTCTTTATTGTCAAAATGGTACCAAGCTTCACGTGATTCCTGTATTGGCTTCTCGATCCCAGGCCCTCAGGTATATTTTACGTGTGATTATGTTTGTGGAACAAGTTGAATGATACCACATTTTATTTGGGATCCAAATGATCCCTTGGTTTCCAGTCACTCACCGTCACAACTCTTTTGATATACTAGCTAACACAAATCTGTTATATCATTTCTTTCACTGAAATTTTGTGGATTGGCTTTTGTACTTACTACAAAGGTTAATTATTTAGCACTTGGTCATATTTCCAGGTACCTACATGTCCGGTGGGGCATAGATTTATCGAATATGGTGGTCTTTACAGGAGAATGTGGGGACACAGACTATGAAGGCTTGTTTGGTGGAGTCCATAAAACTGTGATTCTTAAGGGAGTTGGTATTGAAGCTCGAAAGCTTCATACTAATAGAAACTACCTGTTAGAGGACGTTATACCGTTCAACAATCCCAATATTATTCAAACCAAGGCTTTCGACAGCAACAGCATAAAATCATCATTGGAA

AAACTCGGGGTTCTTTAA

>*LcSPS3*

ATGGCTGGCAACGAGTGGATAAATGGATACTTGGAGGCCATACTGGACAGTGGTGCTGGCGCGGCGGCCATAGAGGACCAGAAGCCTACTCCCGTTAACTTGACGACGGACAGGGGTCATTTCAACCCCACCAAGTATTTCGTGGAGGAGGTGGTTACTGGCGTCGATGAGACTGACCTTTATCGCACTTGGATCAAGGTGGTCGCCACCCGCAACATCCGCGAGCGTAGCTCCAGGCTCGAGAACATGTGCTGGCGCATTTGGCACCTCACCCGCAAGAAGAAACAGGTCTCTCTCCCCCTCTCTCTCTGTGGACTGTGTTTCTCTATATTAATATATTTGTCAGTCTGCATGCATGCATGGAGCTGTATGTATATGTGTATGTATGTGTCTGTGCGCGTATTATTGGCGGTGTCAACAATGCAATGGAGAATTCTGTAAACACGTTTTAATGGCTGTTGGTTGAGTTTCTTGGATGTGTGGCAATTTGACAAGTGGATTTTGTCTATATGTTAATTTCTCCTAGTAAATTGTGAGTTATTATTTTTTTTTTTAGCATGGTTGATGATTATGTTTTTAGCATGGTCTTTTAGATATTAAGCTGTTTTTTTAAGGAGTAATTTTACCTAGCAGATGGTCCCAGATCTCAATTATTAAAAGCAAGATTTTCTGGGGTTATCTAAACAAGAGAGATAACTGGGTTTGCCGAGCTTATACCTGTCCATTCGTGACATTTGTGACTTTTATTGCATGATGTGGGGTTCAGTCAAGCTTTTCTTTATAAATAATGAGTATGCACGTTACAGTTTCTTGTAAATAGTGAATATTGAAGTTTAAGATAAATATTGAATGAGTTGGAGTTCAAGATTATGTGCATATCTTTAAGAAGAGTTTGCATATTTATGTTAATGTACTTAAATACGGACAAGATGGTAGATTTGTGAATTTTTTTATATATGTGCTCATGCACATGCGTGTACGCTCACAATTCGTTAGTTGATTTGACTGTTAAGCAGTCTCTGGTAATCGTCGCATTGAATTTAGTGATCTATATAGATAGGCAGTTGAAATAAACTGTTGTAATCATTGGCATGGAAGTTTCCAAAATTGAGAGGGAATGATTTCCTTTTAGCACTCAGCTCCTGAGTTGAGAAAGCTAATAACATGAGATAATTTATATGTATAATTATAATCAAATTGACTGACTAAAAATGGCTGTCAATGATTTGAATATAGCTGGAGTGGGAGGAAGTACAACGCTTGGCAGACAGGAGATGGGAGCGGGAGCAAGGGCGCAGGGACGTAACTGAAGACATGTCTGAAGACCTGTCAGAAGGTGAAAAGGGAGATGCATTGGCAGAGCTGGTGCAATGTGAGGCTCCAAGGAAAAGTTTCCAACGACAACACTCCAACTTAGAAGTATGGTCGGACGACAAGAAAGAGAAAAAACTCTACATTGTTCTTATCAGGTATGATTCTGTTATACACCTTATTATACTAGGGATCTCCGTTTATGTTGTTTTAGCCATTAAATTGCTTGATCCACATATAACTTATTTTCTAATGGCTATGTTGTCACTGACTTCTCAATATTCAGCAGTTGAACTTTTGAGCTCTGTTTGGGGATGACTTTATGATTATTAAGTTTCAATAGTCGTAGAATGAAACTTATGTCCTTTTTTTCGAACTATATTCAACAGAATAAGATAAATGTATGCTTTGTTTTGAAGTTCAGGTTCAGATAAGACAGAGGATAATGGAATGACTCATGGAGTTGTTAAGCAACTGCTGTAATATGTATAAATTGCAGTTTAATTGTTAGTCCTCCCCCTTTGCTTTGGGCATTTTTTTGTTTTAAATGGATCAGACAGAGAAGTAGGATTAGAAGATAAAAAATGCAATTTGATGTTACCAGATGCAGGGTTTGTAGTATGAAAACAATGCTCTATCTAGAGTAAGCTTTAAAGAACTACGATAAAAGAGCCTCTTTAGGAAACCATTTTATTTTTGCAACATGAATCAAATTATTATGCTATGAACAACATCGAGAACAAAATGCAAAGTGTAATTTATATCCCTTGCACTTGCTTGTTACCTGTACGTATGGCTAGAGCTTATATTTATAAACTTGCTTGCACCATCAATGGCTGTGATTTTCTAGCTAATCGTTAAGAACTTTATATAATTATGCCCAGTTTGCATGGTTTGGTCCGGGGAGATAACATGGAGCTTGGTCGGGATTCTGACACTGGTGGACAGGTATCTAGTGCATTTTGACGTAAGTAAACAACATGACCACTAATTTATCTTTTCTCACTTTACTGCTGTTTTGTGTTTATGAAAAACATATCAGGTCAAATATGTGGTAGAGCTTTCCCGTGCACTTGCAAGGATGCCGGGCGTGTATAGGGTAGATCTCTTTACTCGCCAAGTCTCTTCTCCAGAAGTTGATTGGAGTTATGGGGAGCCAACAGAAATGCTGACGTCTGGTGCCGAAGATGCCGATGGAAATGAAGTGGGCGAGAGCAGTGGGGCATATGTTATTAGGATTCCATTTGGTCCGCGTGACAAGTACCTCCGGAAAGAATTACTGTGGCCCTATATTCAGGAGTTCGTAGATGGAGCGCTTGCCCATATTCTAAATATGTCAAAGGTTTTAGGTGAACAAATTGGCAGGGGCCATCCTGTCTGGCCATATGTGATTCATGGCCACTACGCGGATGCAGGGGATAGTGCTGCTCTTCTTTCTGGTGCTTTGAATGTCCCAATGGTTTTGACTGGACATTCACTAGGCAGAAACAAGCTCGAACAACTTCTTAAGCAGGGACGGCAGTCGACGGAGGATATCAATTCAACATATAAGATTATGCGAAGGATAGAAGCAGAGGAGCTTTCCCTTGATGCTGCTGAACTTGTTATCACGAGTACTAAACAGGAGATTGAAGAGCAATGGGGACTTTATGATGGGTTTGACGTGAAGCTTGAGAAAGTTTTACGTGCTCGTGCTAGACGTGGGGTCAATTGTCATGGCCGATACATGCCCAGGATGGTGGTAAGAATGTGATTTCTATATATATATTTTTTCCAAATCATTGATTTTTTATTCTAGTTGTAAGAGTGGTAGTCAATAAATGGGTGGGAAAATTTTGAATGCATCAACATATACGTTACAATCTGTATTTTTGAATGTGAAACTTTAGATTCCATTATGAGATTTTCAATTAGCACAAACTTCGTATTACAAAATTTCTCTCATAGAGCTTATTTCTAGATGGAAAACGACTTGAGGTCTAATTGAATGCTGGAGTTACTATTATGAATTTAATCCCCTGCTCATGCTTGTAAATTTTGAATTAATGTACTCTTTAACAAAACAAAAAATAGAAATTGAAACAAATATTTAAAGATACATATTTCCGATGCATTAATTCTTGAAATTATCCTTTTCAAGTTCTAAAGTCTAATATTACAGGTTATTCCCCCTGGCATGGACTTCAGCAATGTTGTGGAAGAAGATGCCCCTGAGGCTGAAGGGGAACTTGCAGCTCTTACTGGTAGCAATGATGGATCTTCTCCAAAAGCAGTTCCAGCAATATGGTCCGAAGTGAGTGTCGTATTTGTGTTACACTGTTACTATGCAATGATATTAATTTAATTTATTGTTTCACTACTACATATTTTGGCAGGTGATGCGGTTCCTTACAAATCCCCACAAACCAATGATCTTAGCTTTATCAAGACCTGACCCAAAGAAGAACATGACAACACTTTTGAAGGCCTTTGGAGAGTGCCGTCCCTTAAGAGAGCTTGCTAATCTTGTAAGTGTATTTGTGCAACTACATGTTCACTCAAAGATTACTAAATAATATGCTCAAGCAATCTCTATGCTTTTATGCAGACACTGATAATGGGGAATAGGGATGATATAGAGGAGATGTCTGGAGGAAATGCTAGTGTGCTTACAACAGTATTGAAACTGATTGATAAGTATGACCTCTATGGGCAAGTTGCCTATCCGAAGCATCACAAGCAATCTGATGTTCCAGAAATATACCGACTTGCAGCTAAGACAAAGGTTTGCTGAAGGCCATAATTTTTAAACTAGCTTCATGTGGTCGAATATTGATGATGATCTCAGAGATGAATTGTTACAGGGAGTCTTCATAAATCCGGCACTGGTTGAACCATTTGGGCTTACATTGATTGAGGTTGATTCTGATTCATTACTAGTTTTCATGATGTGATCTTCATGTGCAAGGACGTGGTATGTGATCTATGTTTGTTTGATTGTAGGCAGCAGCTCATGGGCTTCCAATGGTGGCTACTAAAAATGGTGGACCAGTTGACATCCATAGGGTAAGTATGACCTTCATCTATTTGAATCATACGATATTTCTTGTCTACTCACCTGAGGTTCATTAAGTATTGATCCCTTATAAAGGATGTGAAAAGTAAAAATACATGGATCAACCATTTGGTTTAAAATGTCATTAGTTATCTAACCGGTTGCTTTCACTTATTATGGGTTTTAGGCTCTGAACAATGGCCTGCTTGTGGATCCCCATGATCAGCAAGCCATTGCTGATGCACTGCTTAAGTTGGTGTCAGAGAAGAACTTATGGCATGATTGCAGAAAGAATGGTTGGAAAAACATACACCTTTTCTCTTGGCCTGAGCACTGTCGCACTTACTTAACGAGGGTAGCAGCTTGCCGAATGAGGCACCCACAGTGGCAAACTGATACTCCAGGGGACGAGATGGCTGCTGATGAATCGTCTCTTAATGACTCACTGAAGGATGTTCAGGATATGTCACTCAGGCTCTCAGTTGACGGAGACAAACCATCACTGAATGAATCTCTCGACTATACAGCTGCCGCAACGGGTGATCCCGTGCAGGACCAAGTGAAACGAGTTCTAAGCAAGATCAAGCAAACTGAATCCCCTGCAAAGGATAAGGAAGGTGGGAAGAAACTGCTTGAGAATGTGATGAGCAAGTATCCCATGTTGAGGCGGCGCCGTAGATTGATTGTTATTGCTCTTGACTGCTATGATAGCACGGGTGCTCCTGACAAGAAGATGATAAAAATAGTGCAAGATATATTAAAAGCTGTTCGCTCGGACACTCAAACAGCAAGATTATCTGGATTTGCTCTGTCAACAGCAATGCCGCTGTCAGAAACAACAAAGTTCTTAAGTTCAGCAGAAATTCAAGTAAATGAGTTTGATGCTCTGATTTGCAGCAGTGGAGGTGAAGTGTATTATCCAGGTACTTACACGGAAGAGGATGGAAAGCTTTTCCCGGATCCAGACTATGCATCACATATTGACTATCGATGGGGTATTGATGGTCTAAAGAAAACCATTTGGAAGCTGATGAATACAAGTGAAGGTGGAGAGAATTCTGTTCAATCTGGCATCCCCATTCAGGAGGATGAGAAATTGAGCAATGCTCATTGCATTTCATACCTGATTAAAGATCCCAGTAAGGTAATAAAATACAATTTTTACTTCACTATGCAAAGGTATTTGTTTTATTGGCAAGGTTTTATAGGTCTTGGTTTTGCAGGTAAGGCGTGTTGATGATTTGAGGCAGAAGCTCAGGATGCGTGGTCTCCGTTGCCATCCTATGTATTGCAGGAACTCAACGAGAATGCAAATCATTCCTCTTCTAGCCTCTCGAGCACAAGCACTCAGGTACGCATAGATACGATTATTGGCATTCAAATAGTATATATTATTGCCATGGGATGGAAAGTGTGGGTTCATGGTTTCTATAGCACTTGAATACCTATATGTTTAATCAATCCTGGATTAAGTTAAGCTTTTCTACCACCCATAGCCACTTTTAGAGTGTGTTTAGAGAGTGTTAAGACAATGTTTTAAAATAAACTAATAAACAGAACACACGCCTTCAAATGAGACTGGTTTCTAAAACACTTTTAGGATCCTTCCCTAGAGGCCACAGGCAGACAAAAGCTTTAGAAATGTGATGTTAAAAGAAACTGTTTAAGTATGAAACTGTTTAAGTATCGTTTCTATAGTAACCACTAAATCCAAAAATATAAACTATCAGGAAGGACCCAATAATAGTATTTATACTCTAACACATCTCTTCACGTGTAAGGCGGACTGTGGGCTGACAATTGATACACAAGTTCACTATGCTCTGCAGTGTAATTATATAATTAAATTAATAGAATTGGAGTTCCCAGGATTAGAACGTGAGACTGTTAAGTTGACTACATTATGATATTATGTTAATAATCTCTTGAATCAAGTGCTCAAACTATGAGGCACAAGCCCAACAATAGTAGGTGTACACTATCAATTTCTTGCCACTGCAAAGATGTCAAATTGGACTAAGTTACCTGCTTGTTTCCAAACTTGCTGGCAAATCTGTTATCCATTTGCTGTTTATTTAAAGCAAAGTTTGTATGTCAATTCTATCATGAATAAAATCATTCTTTTGAAACTTACTGTGGTGCTTCATGTTGATGTGGTTGAATAGCAGTTCTTGCTGCATTGTTGTATGTCATTCATGCTACATTGGTAGATGTCATGTGATACTGAAAGGAAAAGATAGTCATGTAAATTTCATAGAACTGTCTCAACGTGTCAGAACTTTTTAATTAGCTTGGACATATGGTAGTTCTACTGAACAAAACCGTTTGAATTTAGAACAATGAAATATTTAATACCGAATTTTGATATTTTCAATTTTTTTAGTATAACTTTTAGAAATGAGTGCATCGATTCCATATCAATGGATCTTACTGCAAGCAAGTAGTAAGAACCTTTTTAGGCTCATTATTGATGACATTATCACATTAATATGCCTATCTCAACGTTTTATTTTCACGAAATGTCATTTAATAGTTTGCCAATTACACATACCTCATTGAAGTGAGGCTTTATTGTAATCACTTTCCAAATTGATATATGTTCTTCGCTTCTGATTGATAGGTATCTTTTTGTTCGATGGAGATTGAATGTTGCAAACATGTATGTGGTTCTCGGTGAAAGCGGTGATACAGATTATGAGGAGTTGATATCTGGAACTCATAAGACTTTAATCGTGAAAGGAGCGGTGCAGAAGGGTTCTGAAGAATTGCTCAGAGTTACTGATCTAAGAGAGGACATTGTTCCTAGTGAGAGCCCTCTGATTGCACACATAAATGCGGAGGCCAAAGTCGATGAAATTGCCAATGTTTTAAAGCAAGTCTCCAAAGCTTCTGTAGGAATGTGA

>*LcSPS4*

ATGGCAGGAAATGAGTGGATAAATGGGTACTTGGAAGCAATTTTGGATGTGGGAAGTAGCACAAGAAAAAAATTTGATGGGAAGTTGAAGCTCTCCAAGTTTGAAGAGTCCAAAGCCACTAAAGAAAAAGGAAAGTTGTTTAGTCCCACTAAGTACTTCGTTGAAGAAGTTATTAATAGCGTTGATGAATCTGATCTCCATAGAACTTGGGTCAAGGTTAGTTCATCAGCTCTTTCTATTTCTCTTAAGTCATTTTTTATGTAAACTTTTTTAAGAAATTGTTACATTACAATATAAAATTGGTAACTCTTGAAAGAGGCATGCATATATATATATATATATATGGGGCTTTTATCGATAAAAACGTAAAGCATGGTATATGTTTTAGATCTTTTGCCTCCATTCTCTAAACTCTAGGAAGTCTGTTTCAAAAGAAAAGAGAAAAGGTGGTAATGTGATGTAATTATAAATCAATAAACACAACCGGGAATTTTTTTTTTTTGAAAAAGGAAAAAAGAAAATGAAATGAGCATATATTCCAAGTGTTTATTTTTTCTTTTCTTTTGAAATAATATTCCAAGAGTTTTTTGTGTGGCAGAGTTTGCAAAAAAGATTGATTCTTTTAGTAGGTCCCGTGACGACAATTGGATCTTTAGGGTCCACGCCGATATTAACTTTTTTTTTTCATTTTTTCTTTCCCCATTTGATTTGTTCTCCGCTTTCTATTTTATTTTCCGATTATTAAAATATCTCTAATTTATTTCTTTTTCACACAAAAGCTAGTATATTTATTTTTTTGTTAGTAGGTGTCAAAAGAATAGAACAGAATATTTATTTTATGATTTTAATATTTTACAAACAATAAACACATATAGTCATAACTTAAGTAATTAATACTTTTCATTTACATATTTGGATATGAAAAGGATTTAAATTCTAATGTGCATATTTATCTGTATGGATTGAAATTTATCATCCTTATTGTGGAAGTTACATATCTTTTGATAATATAAAAGTGAATTTTCTAATCACATATGTAATTGGTAAATCCTAAGAAATTTTTAATTGTACATATAATCGATAGATCCTGAGAGCAAGGTAGCTTACTTCATCTTGCAGTTTTATCATAATTTTAAAATAATTTTTTTCGGGAGGCAATGGGCATCATGACCCCCCTTCATTTTGCCACTTCTCAAATAGATTTAAGAACATTAGATTTATACACTTTCAATCGATGAGATTTTACATAATATATGTAATTAACAAATATGAAATTAGCAGCAAGTCTTTAATGCATAAGTTCGAATTTCGATATTATTCTATGTTAAAAATATGAATTATATTTATAAAGGATCTAAGTACGAAGGAATGTTAATCAACAAATTTCTTGTTTCTTTTTTTTTTTTTTGAAACTTCTTGTTTCTTTCTTTTACTGGTGTCAAAAAATTGGTAATTGTATTTGTATATGTATTTAGTTTGATAGTTAGTTAACGTTATTAAATGAAAAGGAGAAAAAGAAAAGAGAAACATGTGATGGTGATGGAGATGGTGATTGTGATTGTGATTGTGATGGGTTCTAAGTGAGAGTAGTAATGGTGGAAGTGAATGCAGGTGATAGCAACAAGAAACTCTCGTGAACGCAGTAACCGGCTCGAGAATATGTGCTGGCGCATTTGGCATCTCACCCGTAAAAAGAGACAGGTATTTCTTTTCTCTTCTCTTTCTTTTTATTGTTTTACTTTTTAATATATATATATATATAAATATATATTACTTATCTAATTAGAATGCTTAAATTTAATAATAACATTTAAGAATTCAATGTTAATCTAGGCTATGAGATGCATAGAATAGAAGAGATTCAATTGTTGTTCGTTGCATGTTTTATGTAAAGTAACTTATAAACATAATAATTTCTTAGTCATCTTAAAATTTTTGTAAAAATCAATCAATATTTATCAATGTAAAATGCATTTTTACGCTGATTTAAATTGATATATACACATCGGTATATATCAATTGATTTAAAAAATTATTTCATAATGAGTATTGGAAAGAAAATCACTCAAACAAAATAAGTTTCATTCATTATGAATCTGATATCGATAAATATTTTAGTATTGTTGTAAGTTAAGAGTCATTTATTTTGATTCAACAACAAGTTATGGCCTAATACTTATTATTAGATTATATAAAATGCTATTTGATGATTACCTTCTTAGAAGGAAGCAAACTAAATTTAAAGAATAGGCTACGAGATGATTAGTTACATTATTGAAACAACAACATAGCTAATCTTTAGACTATAAAGTCAATCACAATAAATTAAGGAAACTCCTTTGTGCTTTTCTCTTAGACTTTCAACACATTTTTAGATGGGCGGTGGGGATGGTGCCTTTTCTGTGTAGTCACTTAAAATAGACTAATTGTTGACAATGCCGAAGATCCACCTACAATTATTTTTATGAGTGTAATATTATTCTACATATTTATAATATGGTGGGTTTAGTTTGATTCTATTAAAATAGGGTTTAGGCCTAACTTTATTAAGACTTTAGGAAAAATTTACACCCATGCTACCTCATTAGTGTTTATCTATATTTGGAAAATTTGCATGAAATTTTATTTCTTAATTAATTATTTAGAAACAAATATTTGAATTATATAAATAGTGATTGATCTTAATATATTTTAATAAATCTATGATTTATTCAACCCAGTATCGGTTTAAAATATAAAATTCCTAGTTAGTTTTAATTTTTAAAGCCTTACTTTATCAATTTAAATTTAAGAAAATATCGATTAATTTGTGAAAGTTAAAATCTGGTTTAGCTTGAAGTGGGTTGTAGTTTAATCTAGTGTTGTAGGATTTATTGATTTTATGTAATTTATCAACTTTTTTCAATACTACAAGCTCTATAGCCACCTAAATTTGGATCATTGCTAAGTCTTTTACCTTTAAATTATATTAGACCATTATAAATTATTAAATATTAATCGACACATAAACAATGGAGAAGATTTTATAAGAACAGAAACTTGAAGTTTTTGACTTACTTTTCTATTAAAATAAATATAATTAGCTTAATATTTATATATATTTTTTTGCAAGAAAATCTACCAATTAATTCAACTAACTGATCGTTAAACACTAAAATTAAACAATTTTGAATTTGATTGACTAATTGTTTGTACGATTTGAATTAAATTTGAACAATTAGTTGCATGTTAGTCGTTATTACAAATTTCTTTTGATGGATGAATGTTTTTTATTAATTAGATTGCATGGGAGGATGCACAAAGGCTTGCAAAGAGACGACTGGAGAGGGAGCAAGGTCGTAACGATGCTGCCGAAGATCTTTCCGAGCTCTCCGAAGGCGAGAAGGAGAAGGGCGATACCAATGTCTCCGAATCTGTCAAGGACATACCGAGAGTCAACTCTGATATGCAAATTTGGTCCGACGTTCATAACTCCGGCAGCCTTTACATTGTCTTGATCAGGTATATATATATATATCTTCAAGTATCTGATAAGCCTTGGTTCTCTTCAAGTATCTTATAAGCCTTGGTTCCCACCATTTTTGTTACACCATATTATATCATATTAATAAATTAGCCTCATAGTACTATTTTAGTTGATAACTATTTGACAAGCTTCACATACCGTATTAAATTATATGATATGAGGGTATATATATGGATAGGATGACTGTGTGACGTCAAATGTTTAATTATATATAAATGACAGATATCAAACAAAGATTTTAAATGATAGATATCAAACAACGATTTTATTGGCAAGAGTAATGAAACAGATCGTAATCTAAATAATTCTAACTAGGTATTATTATGAATTTAAATTTTTTATAGATTCGAGCTTTCATTGAAATATGATTTTTGAAATATTATTATCAAGGTATAGAATAAGGAAGGAATGAATTTTAACCACCTGTATATAATTAATAATGTTGATATTTTATTCTAAAAAAATAATGTTGATATTTTATAAGATATACAGTATTATTTGTTTTTAAATTAAGAGAAACGAAAACTAAAATAAAAGTAATTATTGCTTTCTTAAGTGTTTTTTTTTTAAAATGATTTTTGGCATATCCTCTTAGAAGTTAGAACACATATAAACAAGTTTTAAATTTAACGTACAGCATTGAATTTTGCTTGTATATAGTTTGCATGGATTGGTGCGTGGAGAAAATATGGAGCTTGGAAGAGATTCTGATACTGGTGGACAGGTTGGTGATTGTAATAAATTTATCACAGCTACATTTTTTTTCACTTCTTGTTTTTTCTTGGAAACTAATTTACATACAAATATATAAAAATTCAGGTGAAATATGTTGTTGAACTTGCTCGAGCCTTGGCAAACACAAAGGGTGTGTATCGTGTGGATCTCCTCACTAGACAAATTGCCTCACCGGAGGTCGACTACAGCTACGGTGAGCCCATTGAGATGCTCTCCTGCCCCTCCGACGGCAGCGATAGCTGTGGAGCCTACATCATCCGAATCCCATGTGGTCCTCGTCACAAGTAAATCGCACATTTCTTTGTTTTCATATATTTTACCTTAATATGAGTAAGGTAGCAAAAACGTTATTAAGGATCATGAATATAGCTACAAAAGCATACATATATACACATATCTATATATATATATATAACATAGCAGGAACATCTTTAGATTGTAATATAGATATGGAAACACTTAGTTTTTCTATTTGAAAGTGCTTTCAATATGTCAAAATGGTTTTTAAATGATTCGATTAATTATGTATCATTTTAGACGTTAAACTTGTAAACTAATTGAAAAGTTTGGGATCTTTAATAGGTACTTACCAAAAGAGTCACTATGGCCTCATATTCCTGAATTTGTTGATGGAGCTTTGAGTCACATTGTGAACATGGCAAGGGTACTAGGAGAGCAAGTCAATGGAGGAAAACCAACATGGCCCTATGTGATTCACGGGCACTACGCAGATGCTGGAGAGGTGGCGGCACACCTCTCTGGTGCCTTGAATGTGCCAATGGTACTAACGGGGCACTCATTGGGGCGGAACAAGTTCGAGCAGTTGCTCAAACAAGGGAGGCTTTCTAAGGAGGACATAAATGCAACCTACAAGATCATGAGGAGGATTGAAGCTGAAGAGTTGGGGCTGGATTCTGCTGAAATGGTGGTGACTAGCACAAGGCAGGAGATAGAAGAACAATGGGGGTTGTATGATGGGTTTGATATCAAGTTGGAGAGGAAGCTGAGGGTCAGGAGGCAGCGTGGAGTGAGCTGCCTCGGACGTTACATGCCAAGGATGGTGGTATGTACATTAAATTCAAGACGAATATGACATTTGATTCATGATCTGAAAATACTAAAATGAAACATTAGGGGATATATTAATAACCAACTTTTGTTGCATATCAGGTTATACCACCAGGAATGGACTTTAGCTACGTTAATACAAAAGATTCATTGGAGGGTGATCTGAAATCATTGATTGGCTCTGATAGAACTCAAAGCAAAAGACATCTGCCTCCTATTTGGTCTGAGGTGATCAAATTTTTTACTTCTTTTCTCTTTATCGAAATTAAAAATCTTTATTTCATTTCACATTCTTAAATATATCATTAAGCGAATCATAATTACCTTGTAGATTATGAGATTTTTCACGAATCCACACAAGCCAACCATACTAGCCTTGTCCCGTCCTGACCCGAAAAAGAATGTCACCACATTGCTCAAGGCTTTTGGGGAGTGCCAGCCACTCCGAGAGCTAGCCAACTTGGTAAAAACAATTTGTTTTACTTCAATGACAATTCATATAAATTCAAGCTAACATGCTGACAGATTCATTTCATTGTTATTGCAGACACTAATACTAGGAAACAGAGATGATATTGAAGAGATGTCAAACAGCAGCTCAGTTGTTCTTACTACAGTACTCAAGCTCATTGACAAGTACGACTTGTACGGTCAGGTGGCCTATCCCAAGCATCATAAGCAATCTGAAGTACCTGAAATTTATCGTCTGGCTGCAAAAACAAAGGTATGCTATATGTGGCTTTTTGAATTTGTGAACACATATCTAGCATGATCACACAATGTACAATGTATCTAGATGATAATTGAATAATCAGAAAAGCTAAGGACATTAATAATCAACAACGTATCAACAACATATCAGATCAGATTACCGTCTAAAACTTAACAACACATTAACTCCTTCTCTTCCTTGCAACATTAAATCTGCTAACAGTAATACTTCTATGGCGGCTTTGTATATTGAAGGGAGTTTTCATCAATCCAGCTCTTGTGGAACCATTTGGTCTCACACTCATTGAGGTAAGTATCATTTTTCCCATTCACAAACCACATTTATCATGAATCAATAATTTATGTGCACATATACCAATCTCTTACACTCCATTTCAATGCATTTCTTGCAGGCAGCTGCATATGGTTTACCAATTGTCACCACCAAAAACGGAGGACCTGTGGACATTGTGAAGGTTCTAACTTTCTTTCCCTATAAAAATAGTGATAGTGTCTTAGTTGAAAAATAATGGATCTATCACTCACAATTATGCAGATAACGGAAATTCCTCATGTAACATCCAAACATGTGCCCCAATTTTAGCTAAAGCAGGGCTGAATTGCAGCATACCCTTTGAATTTGACTAGCCTCATAAAATCTATATCACGAACTTGTAAATCATATGTTTCACCAAACTAATAGTGGCAGAGGATTGTGTTTTCTTCAGGCACTTAACAATGGCCTCTTAATTGATCCCCATGATCATAAAGCCATTGCAGATGCCCTATTAAAGCTGGTTGCGGACAAGAACATGTGGTCTGAATGTAGGAAAAATGGCCTCAAGTATATCCACCGCTTTTCATGGACAGAACACTGCCGTAACTACCTCTCCCATGTAGAACACTGCAGGAACCGCCACCCAACAGCCCGTCTTGAGATCATGAAGGTTCCTGAAGAACCAATGAGTGACTCCTTAAAGGATGTGGAAGAACTCTCTTTGAGATTCTCTGTGGATGGAGACTTCAAGCTTAATGGGGAGCTTGATGCAGCAACCAGGCAGAAGAAACTCATTGAAGCCATCACTCAACAGGCTTGTTTCAATGGGAATACAAGTGCCACTCACAGTCCCGGTAGAAGACAAGTTCTATTTGTAATAGCTGTGGATTGCTATGACAGCAATGGTGACACCACGGAGAACTTCCAAGCAGTTATCAAGAATGTAATGAAAGGTGCAGGACTGAGTCTAGGCTTGGGGAAAGTAGGCTTTCTATTGGTGACAGGTTCGAGTTTACGAGAGACCATGGAAGCACTAAGAAGTTGCCCAGTAAACATAGAAGATTTTGATGCATTGATTTGCAATAGTGGAAGTGAAATCTACTATCCATGGCGAGACATGGTGGCTGATTCAGACTTCGAGGCTCATGTGGAGTACAGATGGCCTGGTGAGAATGTGAGATCTATGGTGCCTAGGCTTGCTAGAGTAGAAGATGGAGCTGAGGATGACATCTCTAAGTATGCGATTGCGTGGAGTGCCAGATGCTATTCTTATAACATAAAACCAGCAGCCAAGGTAAGATATCTGTGATCATGTATAAAATCGCCTAGTATAAGAAATGTAGTGTAATGAATATGTACAATTGGCAGGCTCGAAGAGTAGATGATCTTCGTCAAAGGCTTCGAATGAGAGGCATCCGATGCAACATTGTCTTCACACGAGCAGCATCAAGGTTGAATGTAGTGCCATTGTTTGCGTCAAGAATACAAGCTCTAAGGTTAGACCTATCTTATTTCGTACTATTGATCGAATGTAGAGAGACAGTGAAAATTATAACCAACAATGTTTGCATGCATTATGCAGGTATCTATCAATTAGGTGGGGAATAGATCTTTCAAAAATGGTTATGTTTGTGGGCGTAAGAGGAGATACCGACTACGAAAGCCTGATAGCTGGCCTCCATAAGACAATAATTCTAAGAGATGCTGTGGTGTGTGGAAGTGAGAAGCTTGTTCACTGTGAAGATGGTTTCAAAAGCGAAAATGTAGTCCCAGAAGGCAGCAGCAACGTCACCTATGTAGAGGAAGGTTTTGAAGCTCAGAATATC

TCTGCAGCTATAAAGGTTCTTCAGATCAAGTGA
